# Cognitive reflection correlates with behavior on Twitter

**DOI:** 10.1038/s41467-020-20043-0

**Published:** 2021-02-10

**Authors:** Mohsen Mosleh, Gordon Pennycook, Antonio A. Arechar, David G. Rand

**Affiliations:** 1 Science, Innovation, Technology, and Entrepreneurship (SITE) Department, University of Exeter Business School, Exeter, EX4 4PU UK; 2 Sloan School of Management, Massachusetts Institute of Technology, Cambridge, MA 02138 USA; 3 Hill/Levene Schools of Business, University of Regina, Regina, SK S4S 0A2 Canada; 4 Center for Research and Teaching in Economics, CIDE, Aguascalientes, Mexico; 5 Department of Brain and Cognitive Sciences, Massachusetts Institute of Technology, Cambridge, MA 02138 USA; 6 Institute for Data, Systems, and Society, Massachusetts Institute of Technology, Cambridge, MA 02138 USA

**Keywords:** Human behaviour, Human behaviour

## Abstract

We investigate the relationship between individual differences in cognitive reflection and behavior on the social media platform Twitter, using a convenience sample of *N* = 1,901 individuals from Prolific. We find that people who score higher on the Cognitive Reflection Test—a widely used measure of reflective thinking—were more discerning in their social media use, as evidenced by the types and number of accounts followed, and by the reliability of the news sources they shared. Furthermore, a network analysis indicates that the phenomenon of echo chambers, in which discourse is more likely with like-minded others, is not limited to politics: people who scored lower in cognitive reflection tended to follow a set of accounts which are avoided by people who scored higher in cognitive reflection. Our results help to illuminate the drivers of behavior on social media platforms and challenge intuitionist notions that reflective thinking is unimportant for everyday judgment and decision-making.

## Abstract (web summary)

Performance on a cognitive reflection test correlates with a wide range of behaviours in survey studies. Here the authors investigate the relationship between cognitive reflection and some aspects of actual behaviour on social media.

## Introduction

Social media has become a central force in modern life—it is a major channel for social interactions, political communications, and commercial marketing. Social media can have both positive and negative impacts. For example, on the positive side, user-generated content on social media has facilitated social connection by helping friends and relatives who are separated by distance stay abreast of what is happening in each other’s lives^1,2^ and by helping to connect strangers who have similar interests^3^. Social media has also helped to spread awareness of diseases and philanthropic causes (e.g., the ALS ice bucket challenge^4^), helped people in need to generate resources (e.g., crowdfunding for medical bills^5^), and quickly disseminated information during disasters (e.g., Facebook’s “marked safe” tool^6^). However, social media also allows the spread of misinformation^7^ and scams^8^, may facilitate the emergence of echo chambers and political polarization^9,10^, and could be a host for interference and automated propaganda bots^10,11^.

Given the substantial importance of social media in people’s lives, and the wide range of content available therein, it is therefore of scientific and practical interest to understand how people interact with social media, and what influences their decisions to share various types of content and follow different accounts/pages. Prior work in this vein has explored the relationship between social media use and various personality and demographic measures, such as the “Big-Five”^12–14^, the “Dark Triad”^15^, partisanship^16,17^, age^16,17^, and gender^18^.

Here we add to this literature by using a cognitive science lens to explain components of social media engagement across a wide range of content. This also allows us to contribute to an ongoing debate within the cognitive science literature between two competing accounts of the factors that determine people’s beliefs and behaviors. This debate is grounded in dual-process theories, which distinguish reflective or analytic thought from the intuitive responses that emerge autonomously (and often quickly) when an individual is faced with a triggering stimulus^19–22^. One of the key implications of this distinction is that analytic thinking (unlike intuitive processing) is, to some extent, discretionary—that is, people may or may not engage in deliberation and this tendency varies across individuals^20,23^.

Consider the following question: “If you’re running a race and you pass the person in second place, what place are you in?”^24^ The answer that intuitively comes to mind for many people is “first place”; however, second place is the correct answer. This problem illustrates the importance of overriding intuitive responses that seem correct via analytic processing^25–27^. Here we investigate how this individual difference (“cognitive style”) relates to behavior on Twitter. To do so, we measure cognitive style using the Cognitive Reflection Test (CRT)^25^—a set of questions with intuitively compelling but incorrect answers (such as the example above) that is widely used in behavioral economics and psychology to measure the propensity to engage in analytic thinking (and that does not strongly correlate with personality, e.g., “Big Five”^28^).

While there appears to be general agreement surrounding the theoretical utility of dual-process theory (but see refs. ^29,30^), there is a great deal of disagreement about the relative roles of intuition and reflection in people’s everyday lives. It has been famously argued that humans are like an “emotional dog with a rational tail”^31^—that our capacity to reflect is underused in such a way that its primary function is merely to justify our intuitive judgments^32^. Similarly, it has been argued that the main function of human reasoning is argumentation—that is, convincing others that you are correct—rather than truth-seeking per se^33,34^.

The “intuitionist” perspective implies that the real-world function of reasoning is to merely justify and reinforce the beliefs and behaviors that we have learned culturally. Relatedly, it has been argued that the human capacity to reflect actually reduces accuracy by driving polarization around ideological issues^35,36^. This, in turn, implies that whatever variation emerges between individuals on reasoning tasks in a laboratory context is unlikely to be predictive in terms of everyday behaviors for the simple reason that variation on a skill that is ineffective or unimportant should not predict behavior.

However, there is also a growing literature that demonstrates positive everyday consequences of analytic thinking. This “reflectionist” perspective^37^ argues that thinking analytically actually does have a meaningful impact on our beliefs and behaviors and typically does so in a manner that increases accuracy. Evidence for this account comes from laboratory studies where cognitive style is positively associated with a wide range of social phenomena, such as religious disbelief^38,39^, paranormal disbelief^39^, rejection of conspiracist claims^40^, increased acceptance of science^41^, and rejection of pseudo-profound nonsense^42^. More reflective individuals are also less likely to offload their thinking to internet searches^43^. Of particular relevance to the current paper, people who perform better on the CRT are less likely to believe “fake news” stories^44–46^ and they self-report a lower likelihood of sharing such content on social media^45,46^, as well as reporting less trust in unreliable fake news or hyper-partisan news sources^47^. Finally, self-reported actively open-minded thinking style—which is related to, but distinct from cognitive style—was associated with less tweeting but longer tweets, a lower likelihood of having human faces in profile pictures, and subtle differences in language use^48^. Taken together, this body work supports the reflectionist account and suggests that people who perform better on the CRT may differ systematically in their social media behavior from people who perform worse—and in particular, that higher CRT performers may be more discerning (i.e., less likely to follow and share epistemically questionable or facile content).

Crucially, however, this research is almost entirely based on self-reported beliefs and behaviors in survey studies. This is a substantial limitation because the debate between the intuitionist and reflectionist perspectives comes down to the outcomes or consequences of analytic thinking in the context of daily life. The intuitionist perspective dictates that analytic thinking is not particularly important or effective outside of artificial laboratory settings, whereas the reflectionist perspective dictates that analytic thinking is crucial for dictating everyday behaviors.

## Results

Here we investigate the relationship between analytic thinking and naturally occurring behavior on social media, with the goal of distinguishing between these broad accounts of information processing. To do so, we use a hybrid laboratory-field set-up to investigate such differences by linking survey data to actual behavior on Twitter. We recruited a convenience sample of participants (*N* = 1901; 55% female; Median_age_ = 33 years; see Supplementary Tables 1 and 2 and Supplementary Fig. 1 for further descriptive statistics) who completed the CRT questions and provided their Twitter username. We then used the Twitter API to pull public information from the users’ profiles on Twitter, allowing us to investigate the relationship between a user’s CRT score and three main dimensions of their “digital fingerprint”: basic characteristics of their profile, the accounts they follow, and the contents of their tweets.

In the main text, we report relationships between measures of interest and *z*-scored CRT score (proportion of correct answers given to CRT questions) as the main independent variable, as well as *z*-scored age, gender (0 = male, 1 = female), ethnicity (0 = non-white, 1 = white), political ideology (1 = strong liberal, 5 = strong conservative), US residency (0 = non-US, 1 = US), education level (0 = less than college degree, 1 = college degree or higher), income (1 = lowest income group in the participant’s country, 10 = highest income group in the participant’s country), and time to complete the survey (log-transformed time to complete the survey, in seconds). Furthermore, because the Twitter API only allows us to collect the 3200 most recent tweets, the age of the retrieved tweets may be lower for tweets from more active users; therefore, to avoid temporal confounds, all analyses of tweets include month fixed effects (i.e., dummies for each year–month combination). See Supplementary Tables 1–22 for all detailed models and also accounting for multiple comparisons using either the Bonferroni–Holm correction^49^ or maintaining a 5% false discovery rate using the Benjamini–Hochberg procedure^50^.

### Profile characteristics

We begin by examining the relationship between CRT and basic profile features: number of accounts followed, number of followers, total number of tweets, number of tweets in the past 2 weeks, number of favorited tweets, number of lists, and number of days on Twitter. As each of these quantities is an overdispersed count variable (see Supplementary Table 3), we use negative binomial regression to predict each quantity, taking CRT as the main independent variable, as well as users’ demographics. We find that subjects who scored higher on the CRT follow significantly fewer other accounts (incidence rate ratio = 0.867, *p* = 0.001). This is some first suggestive evidence of higher CRT users being more discerning, in that they follow fewer accounts (and thus expose themselves to less content). But, of course, the specific accounts they follow (discussed below) are much more relevant for following discernment than the total number. The relationship between CRT and all other profile characteristics was non-significant (*p* > 0.10 for all; see Supplementary Tables 4–10 for full regression tables). This includes other potential measures of being discerning, such as tweet count and number of favorites. Thus our analysis of profile characteristics is overall somewhat agnostic regarding the connection between CRT and discernment.

Additionally, we find age, female gender, and white ethnicity are significantly positively related to the number of accounts followed by the user; age, female gender, white ethnicity, and higher education are significantly positively related, and political conservativism and time to complete the survey are significantly negatively related to the user’s number of followers; female gender and white ethnicity are significantly positively related to the user’s number of tweets; female gender is significantly positively related and political conservativism and time to compete survey are significantly negatively related to the user’s number of favorites; age, female gender, white ethnicity, and higher education are significantly positively related to the user’s number of listed accounts; US residency, income, and time to complete the survey are significantly negatively related to the user’s number of tweets in the past 2 weeks; and age, female gender, and white ethnicity are significantly positively related and political conservativism and time to complete the survey are significantly negatively related to the number of days since the user account was created on Twitter. See Table 1 for details.

**Table 1:** Relationship between users’ characteristics and their Twitter profile characteristics.

	Followed count	Followers count	Tweets count	Favorites count	Listed count	Tweets in the past 2 weeks	Days on Twitter
	IRR (SE)	IRR (SE)	IRR (SE)	IRR (SE)	IRR (SE)	IRR (SE)	IRR (SE)
CRT	0.867** **(0.036)**	0.965 (0.045)	0.894 (0.090)	1.099 (0.082)	0.891 (0.098)	0.865 (0.096)	1.016 (0.011)
Age	1.189*** **(0.045)**	1.143* **(0.065**)	1.05 (0.081)	0.84 (0.087)	1.569*** **(0.131)**	1.175 (0.129)	1.096*** **(0.011)**
Gender (female)	1.144** **(0.049)**	1.129* **(0.059)**	1.423*** **(0.10)**	1.269** **(0.10)**	1.464*** **(0.113)**	1.155 (0.109)	1.033** **(0.011)**
Ethnicity (white)	1.129** **(0.051)**	1.176** **(0.062)**	1.215** **(0.075)**	1.022 (0.079)	1.546*** **(0.127)**	1.144 (0.096)	1.031* **(0.013)**
Political ideology (conservatism)	0.954 (0.046)	0.84** **(0.054)**	0.849 (0.087)	0.714*** **(0.059)**	0.879 (0.108)	0.962 (0.121)	0.967** **(0.01)**
US residency	0.950 (0.046)	0.984 (0.064)	0.981 (0.064)	1.038 (0.076)	1.178 (0.131)	0.83* **(0.062)**	1.001 (0.011)
Education (college degree)	1.011 (0.043)	1.124* **(0.057)**	0.976 (0.078)	0.878 (0.065)	1.198* **(0.096)**	0.956 (0.094)	1.01 (0.011)
Income	0.958 (0.042)	1.018 (0.049)	0.887 (0.067)	0.87 (0.074)	1.015 (0.089)	0.746** **(0.067)**	0.983 (0.011)
Log (time to complete the survey)	0.966 (0.048)	0.965* **(0.045)**	0.873 (0.093)	0.882* **(0.056)**	0.885 (0.122)	0.757* **(0.101)**	0.962** **(0.011)**

Results are generated using a negative binomial regression model. Variables are coded as follows: gender (0 = male, 1 = female), ethnicity (0 = non-white, 1 = white), political ideology (1 = strong liberal, 5 = strong conservative), US residency (0 = non-US, 1 = US), education level (0 = less than college degree, 1 = college degree or higher), income (1 = lowest income group in the participant’s country, 10 = highest income group in the participant’s country), and time to complete the survey (log-transformed time to complete the survey, in seconds). *p* values are reported using two-tailed *z*-test and without adjustment for multi-comparison (*p < 0.05, **p < 0.01, ***p < 0.001; see Supplementary Tables 4–10 for exact *p* values and adjustment for multi-comparisons). Statistically significant results are shown in bold.

### Accounts followed

**Fig. 1:**
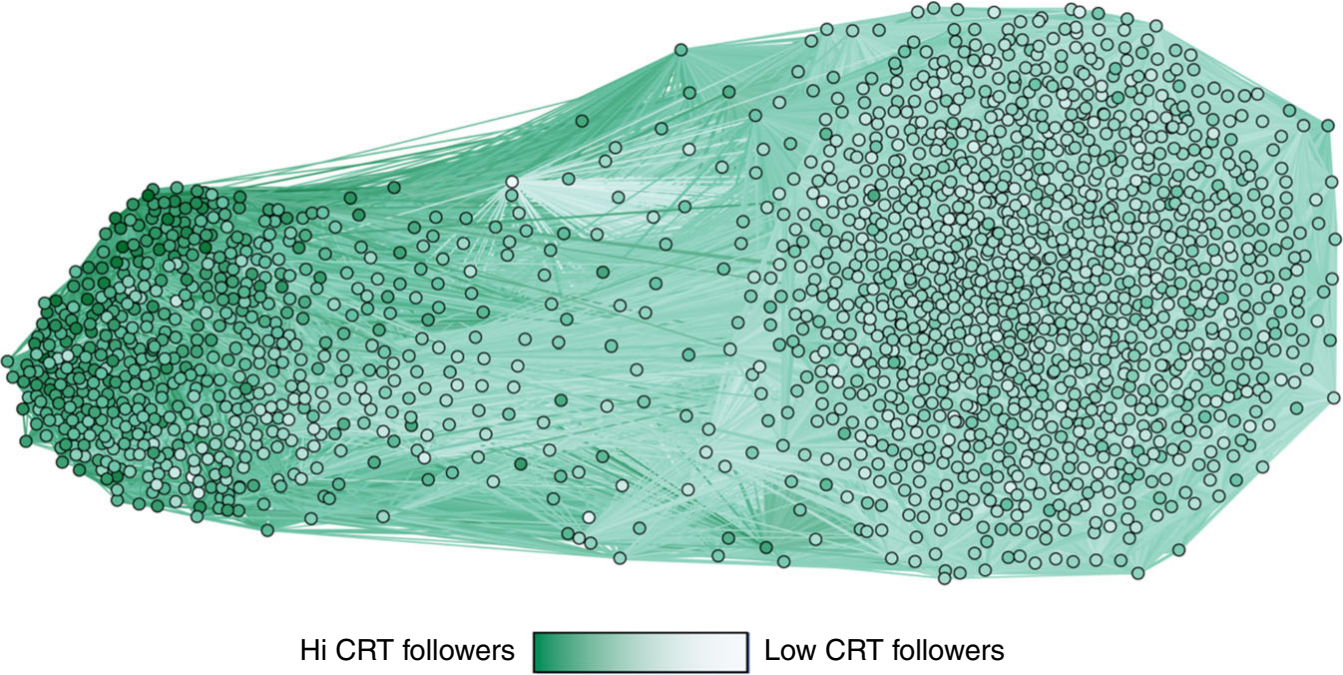
Co-follower network. Nodes represent Twitter accounts followed by at least 25 users in our dataset and edges are weighted based on the number of followers in common. The intensity of color of each node shows the average CRT score of its followers (darker = higher CRT score). Nodes are positions using directed-force layout on the weighted network (edges with weight <5 are not shown for visualization purposes). Visualization depicts two distinct communities that differ in the CRT scores of their followers.

Following up on the observation that higher CRT participants followed significantly fewer accounts, we next examine which accounts are followed by participants who scored lower versus those who scored higher on the CRT—that is, we examine how CRT relates to which types of content users consume on Twitter (the accounts one follows form a good proxy for the content one is exposed to^17^). Specifically, we assess structural differences between the accounts followed by the users given their CRT. To do so, we construct the co-follower network: each node in the network represents a Twitter account that is followed by at least 25 participants in our dataset (1860 nodes; results are robust to using thresholds other than 25, see Supplementary Tables 11 and 12), and the edge between two given nodes is weighted by the number of participants in our dataset that follow both nodes (Fig. 1). Community detection analysis^51^ on the co-follower network reveals two distinct clusters of accounts (Fig. 1). Table 2 shows the 10 accounts in each cluster that are followed by the largest number of our participants. The clusters differ substantially in the cognitive style of their followers (Cohen’s *d* = 1.66; cluster 1: mean follower CRT = 0.515, SD of mean follower CRT = 0.075, fraction of nodes = 0.35; cluster 2: mean follower CRT = 0.419, SD of mean follower CRT = 0.032, fraction of nodes = 0.65). Furthermore, the average CRT of the followers of a given account is a highly significant predictor of which community that account belongs to (logistic regression predicting membership in cluster 2, odds ratio (OR) = 0.545, p = 0.004), such that a one standard deviation decrease in followers’ average CRT score is associated with an 83.5% increase in the odds of an account being in the low CRT cluster. This finding is robust to varying the follower threshold in the community detection algorithm—regardless of the threshold chosen, there are always at least two clusters with a significant difference in average CRT of followers (see Supplementary Tables 11 and 12).

These results are striking, insomuch as they suggest the existence of “cognitive echo chambers” on social media, albeit in an asymmetric fashion: we see a set of accounts (those in cluster 2) that are mostly followed by users who scored lower on the CRT but avoided by users who scored higher on the CRT. Accounts in cluster 1, conversely, are followed by users who both scored high and low on the CRT (see Supplementary Figs. 2 and 3 for distribution of followers’ CRT in the clusters within the co-follower network). Interestingly, the cluster of accounts that are preferentially followed by users who scored lower on the CRT is roughly twice as large as the cluster of accounts that are followed by users who scored both lower and higher on the CRT (1209 versus 651 accounts).

**Table 2:** Top accounts in each cluster within the co-follower networks.

Cluster 1		Cluster 2	
Account	Followers’ mean CRT score	Account	Followers’ mean CRT score
barackobama	0.543	aldiuk	0.471
stephenfry	0.567	poundland	0.446
bbcbreaking	0.542	argos_online	0.450
realdonaldtrump	0.526	bmstores	0.443
jk_rowling	0.535	morrisons	0.460
rickygervais	0.566	nextofficial	0.427
theellenshow	0.526	lovewilko	0.435
amazonuk	0.513	superdrug	0.381
nasa	0.630	ukmagicfreebies	0.399
twitter	0.493	top_cashback	0.448

For each cluster, the table shows the 10 accounts in each cluster with the largest number of followers amonst our participants, along with the mean CRT score of each account’s followers.

We also find that followers’ average age, fraction of female followers, and fraction of followers with at least a college degree are significantly positively related to membership in the low-CRT cluster, whereas followers’ average income and time to complete the survey are significantly negatively related to membership in the low-CRT cluster in the co-followers network (Table 3). However, unlike all other analyses we report in this paper, many of the variables in this co-follower network-based model are highly correlated with each other because of the aggregated nature of the co-follower data (e.g., numerous combinations of followers’ mean CRT, education, income, gender, and ethnicity have correlations of *r* > 0.6). As a result, the various demographic correlations should be interpreted with caution. Critically for our main question of interest, we continue to find that the average CRT of the followers of a given account is a highly significant and strong predictor of which community that account belongs to when using a model with only CRT and no demographic controls (logistic regression, OR = 0.090, *p* < 0.001).

**Table 3:** Users’ characteristics and clusters in co-followers’ network, quality of content, and tweeting from news websites.

	(a) Membership in cluster 2	(b) Tweeted news websites?	(c) Quality of news sites shared
	OR (SE)	OR (SE)	*β* (SE)
CRT	0.545** **(0.208)**	1.135* **(0.050)**	0.078* **(0.034)**
Age	3.463*** **(0.151)**	1.389*** **(0.051)**	−0.013 (0.038)
Gender (female)	16.38*** **(0.294)**	1.163** **(0.050)**	0.001 (0.031)
Ethnicity (white)	1.014 (0.239)	1.026 (0.051)	−0.004 (0.04)
Political ideology (conservatism)	1.308 (0.185)	0.784*** **(0.050)**	−0.177*** **(0.046)**
US residency	0.923 (0.342)	1.006 (0.049)	−0.078* **(0.036)**
Education (college degree)	1.498* **(0.193)**	1.076 (0.051)	0.055 (0.039)
Income	0.230*** **(0.232)**	0.96 (0.051)	0.025 (0.038)
Log (time to complete the survey)	0.332*** **(0.157)**	0.857** **(0.051)**	−0.07* **(0.031)**

(a) Logistic regression predicting which cluster the account belongs to using the average characteristics of their followers in our sample (threshold of number of followers from our sample *K* = 25). (b) Logistic regression predicting if the user tweeted from news websites. (c) Linear regression predicting quality of news sources contained in each tweet based on the users’ characteristics, including month fixed effects and clustering standard errors on user. Variables coded as follows: gender (0 = male, 1 = female), ethnicity (0 = non-white, 1 = white), political ideology (1 = strong liberal, 5 = strong conservative), US residency (0 = non-US, 1 = US), education level (0 = less than college degree, 1 = college degree or higher), income (1 = lowest income group in the participant’s country, 10 = highest income group in the participant’s country), and time to complete the survey (log-transformed time to complete the survey, in seconds). Variables used in (a) are aggregated over followers characteristics. *p* values are reported using two-tailed *z*-test for (a) and (b) and using two-tailed *t* test for (c) (*p < 0.05, **p < 0.01, ***p < 0.001; see Supplementary Tables 11–14 for exact *p* values and detailed statistical analysis). Statistically significant results are shown in bold.

### Contents of tweets

Finally, we shift from the accounts that users follow (and thus the content they consume) to the content users create and/or distribute themselves: their tweets (1619 subjects had accessible public tweets on their timeline, generating a total of 1,871,963 tweets).

We begin by investigating the sharing of news. To do so, we focus on tweets or retweets containing links to one of the 60 news websites whose trustworthiness was rated by professional fact-checkers in previous work^47^; these news sites span a wide range of information quality, from entirely fabricated “fake news” sites to hyper-partisan sites that present misleading coverage of events that did actually occur to reputable mainstream news sources. For our analysis, we extracted and unshortened all URLs in all tweets and collected any tweets containing links to one of the 60 sites (728 users tweeted at least one link from one of these sites, with 11,295 tweets in total).

First, we look at the relationship between CRT and whether a user tweeted any links to these news sites at all. We perform a logistic regression predicting whether a user from our sample tweeted at least one link from the 60 news websites. We find users who scored higher on the CRT are significantly more likely to tweet links to news websites (OR = 1.135, *p* = 0.011). This provides a first piece of evidence that users who scored higher on the CRT are more likely to tweet about weightier subjects, namely the news.

We also find age and female gender are significantly positively related, whereas political conservativism and time to complete the survey are significantly negatively related, to likelihood of tweeting from news websites (Table 3).

**Fig. 2:**
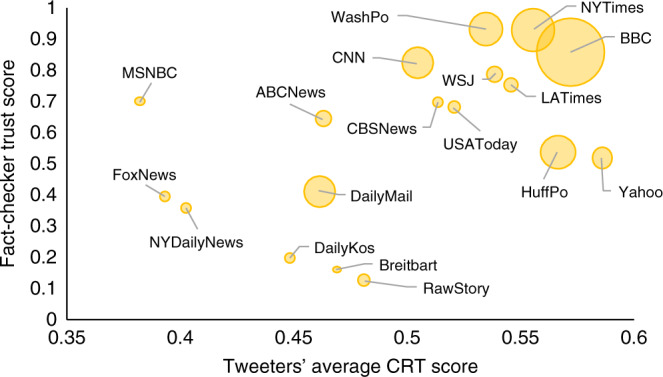
Fact checker trust score of shared news sources versus average CRT score of users. Each dot represents an outlet shared by users in our sample on Twitter. The size of the dots represents the number of observations. For clarity, we show outlets that have been shared at least 50 times by the users.

For those users who tweeted links to at least one of the 60 sites, we then perform a linear regression predicting the trustworthiness of the tweeted news source based on the CRT score of the user who shared the link, with robust standard errors clustered on user and controlling for demographics and month fixed effects. Doing so finds a positive correlation between CRT score and trustworthiness of shared news sources (*β* = 0.078, *p* = 0.019; Fig. 2). For example, higher CRT users were more likely to retweet links to the BBC (OR = 1.232, *p* < 0.001) (which is highly trusted by professional fact-checkers) and less likely to retweet links to the Daily Mail (OR = 0.787, *p* < 0.001) (which is untrusted by professional fact-checkers); these sites are particularly common in our dataset because a plurality of our participants were from the United Kingdom. We also find that political conservativism, US residency, and time to complete the survey are significantly negatively related to the quality of content shared by users (Table 3). See Supplementary Tables 13 and 14 for statistical details.

**Fig. 3:**
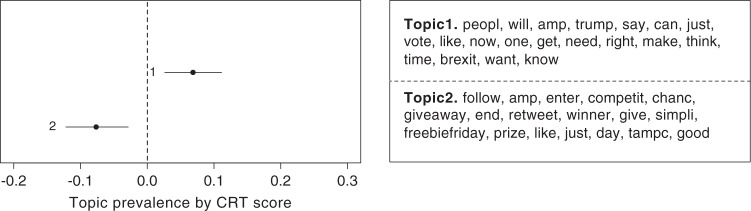
Difference in topic proportion against CRT score of users. Topic 1 related to political engagement is positively correlated with CRT score and Topic 2 involving “get rich quick” schemes is negatively correlated with CRT score.

Next, we examine the topics people tweeted about using Structural Topic Modeling^52^, a general framework for topic modeling with document-level covariate information. We analyzed all tweets and retweets written in English by users in our dataset whose timeline was accessible and who had at least 10 (re)tweets in English (1424 users). For each user, we merged all tweets from the timeline as a document and used the user CRT score as the covariate for the topic modeling. We found that two particular topics are consistently correlated with high versus low CRT scores of users: a topic involving politics (e.g., “people,” “vote,” “trump,” “brexit”) was positively correlated with CRT and a topic involving “get rich quick” schemes (e.g., “win,” “enter,” “chance,” “giveaway,” “prize”) was negatively correlated with CRT. Figure 3 shows the difference in topic prevalence for each topic against the users’ CRT score for a seven-topic model (our results are robust to the choice of the number of topics; see Supplementary Table 15).

Finally, we examined the language used in the tweets at the level of individual words. To do so, we employed the Linguistic Inquiry Word Count (LIWC; a psychologically validated set of word dictionaries^53^) approach to test how CRT scores related to the probability of a user’s tweets containing words in various LIWC categories. Specifically, if people who do well on the CRT are more likely to engage in thinking (insight) to override (inhibit) their intuitive (often emotional) responses, then we might expect positive correlations between CRT and the use of insight and inhibition words and negative correlations between CRT and the use of positive and negative emotion words. Of course, this presupposes that using insight- and inhibition-related words is indicative of engaging in deliberation, while using positive and negative emotion words is indicative of experiencing positive and negative emotion. Although this is commonly assumed by many scholars, it is clearly a substantial inferential leap. Thus these particular results should be interpreted with some caution.

**Fig. 4:**
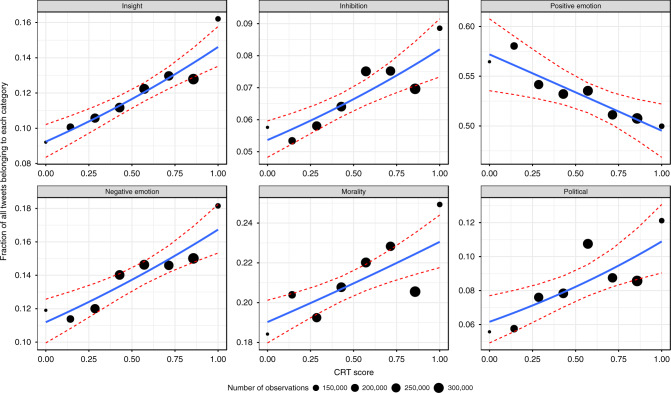
Word categories versus CRT score. For each word category and CRT score, dots represent the fraction of all tweets that have at least one word from that category. The size of the dots shows the number of tweets. Lines represent the relationship between word categories and CRT score using weighted least-square estimation. Red lines show 95% confidence internal based on the logistic regression model fitted on individual observations. Only the relationships between CRT score and “Insight,” “Inhibition,” “Negative emotion,” “Morality,” and “Political” are significant when controlling for demographics.

For each word category, we use a separate logistic regression predicting the presence of that word category in a given tweet based on the tweeting user’s CRT as the main independent variable, controlling for users’ demographics and month fixed effects, with standard errors clustered on user (see Supplementary Tables 16–22 for detailed models). Figure 4 shows the fraction of all tweets belonging to each category as a function of the users’ CRT score. To give a better sense of what specific words are driving these relationships, for each word category with a significant CRT correlation, Table 4 shows the ten words with the largest difference in frequency between low and high CRT subjects (using median split).

**Table 4:** Distinctive usage of words by users who scored high versus low on the CRT.

Insight	Inhibition	Positive emotion	Negative emotion	Moral	Political
think	stop	like	f?ckin*	just	trump
know	keep	well*	problem*	should	vote
feel	conserv*	sure*	sh?t*	right	government
thought	protect*	better	horr*	help	job
idea	wait	thank	f?ck	order*	election
mean	safe*	great	fail*	leader*	black
seems	hold*	hope	sorry	protect*	political
means	control*	party*	numb*	class	rights
found	ignor*	interest*	weird*	nation*	law
understand	refus*	please*	worr*	rights	media

For each category that is positively or significantly associated with CRT, shown are the words that have the highest difference in frequency between users who scored high and those who scored low on the CRT (based on a median split of CRT scores). Asterisk (*) represents every combination of characters that immediately comes after.

As predicted based on the conceptualization of CRT as measuring deliberativeness, we find that users with higher CRT scores are more likely to use words associated with insight (OR = 1.138, *p* < 0.001) and inhibition (OR = 1.133, *p* < 0.001). Contrary to our predictions, however, we found no significant correlation between CRT and use of positive emotional words (OR = 0.966, *p* = 0.235) and a significant positive correlation between CRT and use of negative emotion words (OR = 1.124, *p* < 0.001). It is unclear how exactly to interpret these contradictory results. The inconsistent pattern of correlation may reflect the limitations of dictionary-based methods for assessing the use of cognitive processes.

We also investigated the relationship between CRT and non-cognitive word categories where the connection between word use and interpretation is more straightforward. We explored the relationship between CRT and the use of words related to morality, as previous work has shown that CRT is associated with different moral values^54,55^, judgments^56^, and behaviors^57–59^, but has not examined the relationship between CRT and engagement with morality more generally. And we looked at the relationship between CRT and use of words related to politics (using the dictionary of words suggested by ref. ^60^), as prior work has found a link between CRT and political engagement^61^. We found a significant positive correlation with words related to morality (OR = 1.078, *p* < 0.001) and a significant positive correlation with use of political words (OR = 1.167, *p* = 0.006). As a related dictionary validation test, we also examine how political extremity (e.g., distance from the scale midpoint for the partisanship measure) relates to producing tweets with political language. We found that political extremity is significantly and positively related to the use of political words (OR = 1.235, *p* = 0.019; see Supplementary Tables 16–22 for details). Finally, we had also planned to investigate the link between CRT and religious words, based on prior work linking CRT to reduced belief in God^38^. However, we found that use of religious words was not significantly associated with participants’ self-reported belief in God (OR = 1.022, *p* = 0.33). This raises questions about the validity of the religious word dictionary as an index of religious belief and negates any expectation of a relationship between religious words and CRT (we note that, as in the past work, CRT was negatively related to self-reported belief in God; *β* = −0.11, *p* < 0.001).

Additionally, we find that political conservatism is significantly negatively related, and having a college degree is significantly positively related, to use of insight words; that age, US residency, and time to complete the survey are significantly positively related, and political conservatism is significantly negatively related, to use of inhibition words; that age, female gender, white ethnicity, and political conservatism are significantly positively related, and US residency, income, and time to complete the survey are significantly negatively related, to use of positive emotion words; that US residency is significantly positively related, and age, white ethnicity, and political conservatism are significantly negatively related, to use of negative emotion words; that age is significantly positively related, and political conservatism is significantly negatively related, to use of moral words; and that age, US residency, and time to complete the survey are significantly positively related, and female gender, white ethnicity, and political conservatism are significantly negatively related, to use of political words (see Table 5 for relation between word categories and CRT and other users’ characteristics).

**Table 5:** Relationship between users’ characteristics and the use of words in different LIWC categories in tweets.

	Insight	Inhibition	Positive emotion	Negative emotion	Moral	Political
	OR (SE)	OR (SE)	OR (SE)	OR (SE)	OR (SE)	OR (SE)
CRT	1.138*** **(0.025)**	1.133*** **(0.028)**	0.966 (0.029)	1.124*** **(0.029)**	1.078*** **(0.017)**	1.167** **(0.056)**
Age	0.955 (0.026)	1.100** **(0.031)**	1.118*** **(0.028)**	0.933* **(0.031)**	1.081*** **(0.021)**	1.32*** **(0.062)**
Gender (female)	0.973 (0.023)	0.991 (0.03)	1.200*** **(0.025)**	0.953 (0.026)	1.018 (0.02)	0.882* **(0.059)**
Ethnicity (white)	0.985 (0.018)	0.966 (0.018)	1.068** **(0.02)**	0.954* **(0.02)**	0.964 (0.021)	0.900* **(0.041)**
Political ideology (conservatism)	0.880*** **(0.027)**	0.893*** **(0.032)**	1.079* **(0.03)**	0.862*** **(0.03)**	0.945** **(0.018)**	0.777*** **(0.071)**
US residency	1.032 (0.02)	1.074** **(0.023)**	0.924*** **(0.022)**	1.056* **(0.024)**	1.022 (0.02)	1.245*** **(0.053)**
Education (college degree)	1.050* **(0.025)**	0.991 (0.034)	0.961 (0.028)	1.013 (0.028)	1.012 (0.019)	0.994 (0.059)
Income	0.995 (0.026)	1.006 (0.039)	0.945* **(0.027)**	0.999 (0.029)	0.989 (0.021)	0.970 (0.057)
Log (time to complete the survey)	1.047 (0.026)	1.06* **(0.028)**	0.935* **(0.028)**	1.033 (0.028)	1.015 (0.018)	1.153** **(0.046)**

Results are generated using logistic regression model predicting if each tweet has at least one word from a given category based on users’ characteristics, including month fixed effects and clustering standard errors on user. Variables coded as follows: gender (0 = male, 1 = female), ethnicity (0 = non-white, 1 = white), political ideology (1 = strong liberal, 5 = strong conservative), US residency (0 = non-US, 1 = US), education level (0 = less than college degree, 1 = college degree or higher), income (1 = lowest income group in the participant’s country, 10 = highest income group in the participant’s country), and time to complete the survey (log-transformed time to complete the survey, in seconds). *p* values are reported using two-tailed *z*-test and without adjustment for multi-comparison (*p < 0.05, **p < 0.01, ***p < 0.001; see Supplementary Tables 16–21 for exact *p* values and adjustment for multi-comparisons). Statistically significant results are shown in bold.

## Discussion

Together, these results paint a fairly consistent picture. People in our sample who engaged in more cognitive reflection were more discerning in their social media use: they followed fewer accounts, shared higher quality content from more reliable sources, and tweeted about weightier subjects (in particular, politics). These results have numerous implications.

Returning to the debate between those who have claimed a limited role for cognitive reflection in determining everyday behaviors (intuitionists) and those who emphasize the importance of the (perhaps distinctly) human capacity to use reflection to override intuitions (reflectionists), the results are plainly more consistent with the latter perspective. We find that reflective thinking (as measured in our survey study) is associated with a wide range of naturally occurring social media behaviors. This provides the strongest evidence to date for the consequences of analytic thinking for everyday behaviors: If humans were so dominated by their intuitions and emotions (“emotional dogs with rational tails”), then variation in people’s tendency to reason should not be particularly important for understanding their everyday behaviors. Plainly, this is not the case. Furthermore, each of these associations has important theoretical implications in their own right that we will now enumerate; together, they paint a consistent picture of reflective thinking as an important positive force in judgment and decision-making outside of the laboratory.

One line of prior work that the current results bear on has to do with media truth discernment. Past work has shown that people who are more analytic and reflective are better at identifying true versus false news headlines, regardless of whether the headlines align with their ideology (e.g., refs. ^44,45^). However, these studies have relied entirely on survey experiments, where participant responses may be driven by experimenter demand effects or expressive responding. Additionally, in these experiments, participants judge a comparatively small set of headlines (pre-selected by the experimenters to be balanced on partisanship and veracity). Thus these prior results may be idiosyncratic to the specific headlines (or approach for selecting headlines) used in designing the survey. Furthermore, these studies have focused on contrasting true headlines with blatantly false headlines (which may be comparatively rare outside the laboratory^16,17^), rather than articles that are misleading but not entirely false (e.g., hyper-partisan biased reporting of events that actually occurred^47^). Thus the results may not generalize to the kinds of misinformation more typically encountered online. Finally, these studies have mostly focused on judgments of accuracy, rather than sharing decisions. Thus, whether these previously documented associations extended to actual sharing in the naturally occurring social media environment is an open question—particularly given that the social media context may be more likely to activate a political identity (as opposed to accuracy or truth) focus^62,63^. Yet, despite these numerous reasons to think that prior findings may not generalize outside the survey context, we do indeed find that participants who perform better on the CRT share news from higher-quality news sources. This observation substantially extends prior support for a positive role of reasoning in news media truth discernment.

Our results are also relevant in similar ways for prior work regarding the role of cognitive sophistication in political engagement. Prior evidence using survey experiments suggests that people who are more cognitively sophisticated (e.g., those who score higher on the CRT, more educated, higher political knowledge) show higher rates of engagement with politics. However, it has also been suggested that this relationship may be the result of social desirability bias, such that more cognitively sophisticated people simply over-report political engagement to please the experimenter^64,65^. Our results, however, suggest that more reflective people are indeed actually more engaged with politics on social media. This supports the inference that analytic thinking is associated with increased political engagement.

More broadly, cognitive reflection has been associated with lower gullibility—that is, less acceptance of a large range of epistemically suspect beliefs (such as conspiracy theories, paranormal claims, etc.—see ref. ^20^ for a review), including decreased susceptibility to pseudo-profound nonsense^42^. Again, however, these findings are rooted in survey evidence and not real-world behavior and could reflect socially desirable responding. Here we find that low CRT is associated with increased following of and tweeting about money-making scams and get-rich-quick schemes. This supports the conclusion that more intuitive people may indeed be more gullible.

One of the most intriguing results that we uncovered was the clustering of accounts followed by participants who scored lower versus higher on the CRT. In particular, there was a large cluster of accounts that were predominantly followed by participants who scored lower on the CRT—fully two-thirds of the accounts followed by at least 25 of our participants were in this lower-CRT cluster. This observation is particularly interesting in the context of the extensive discussion of partisan echo chambers, in which supporters of the same party are much more likely to interact with co-partisans^9,10^. Our network analysis indicates that the phenomenon of echo chambers is not limited to politics: the cognitive echo chambers we observe have potentially important implications for how information flows through social media. Furthermore, it is likely that cognitive echo chambers are not confined to social media—future work should investigate this phenomenon more broadly. Relatedly, the clustering that we observe in the co-follower network relates to the extensive theoretical literature on dynamic social networks, and in particular the emergence of clustering in agents’ cognitive style^66,67^. It would be fruitful for future theoretical work to model cognition and networks in the context of co-follower networks, rather than the direct connections considered in past work. Future work should also use field experiments examining link formation and reciprocity on social media^68^ to test for causal effects of shared cognitive style on following behavior.

There are, of course, important limitations of the present work. Most notably, we were only able to consider the Twitter activity of a tiny subset of all users on the platform. Thus it is important for future work to examine how our results generalize to other more representative sets of users—and in particular to users who did not opt into a survey experiment. One potential approach that may be fruitful in this endeavor is training a machine learning model to estimate users’ CRT scores based on their social media activity. Relatedly, it will be important to test how the results generalize to other social media platforms (e.g., Facebook, LinkedIn) and to users from non-Western cultures (e.g., Weibo, WeChat). Additionally, the dictionary-based approach that we used to analyze the language content of the tweets, although widely used in the social sciences, is limited in terms of measuring complex psychological constructs based on usage of single words. Future work should also examine how the results obtained here generalize to other measures of cognitive sophistication beyond the CRT.

In sum, here we have shed light on social media behavior through the lens of cognitive science. We have provided evidence that one’s extent of analytic thinking predicts a wide range of social media behaviors. These results meaningfully extend prior survey studies, demonstrating that analytic thinking plays an important role outside the laboratory. This reinforces the claim that the human capacity to reason is hugely consequential and something that should be cultivated and improved rather than ignored. Research findings that highlight surprising impacts of intuitions, emotions, gut feelings, or implicit biases should be interpreted in light of our findings that explicit reasoning remains central to the human condition.

## Methods

Our study was approved, and informed consent was waived, by the Yale Human Subjects Committee, IRB Protocol # 2000022539.

### Participants

We used a non-representative international convenience sample and included controls for demographic features. We recruited participants via Prolific^69^, a subject pool for online experiments that consists of mostly UK- and US-based individuals. We used a feature on Prolific to selectively recruit participants who self-reported using Twitter on a regular basis. We recruited 2010 participants from June 15, 2018 to June 20, 2018. Twitter IDs were provided by participants at the beginning of the study. However, some participants entered obviously fake Twitter IDs—for example, the accounts of celebrities. To screen out such accounts, we excluded accounts with follower counts above the 95th percentile in our dataset. We had complete data and usable Twitter IDs for 1901 users (55% female, Median_age_ = 33, 43% UK residents, 18% US residents, and the rest mostly from Canada, Spain, Italy, and Portugal; see Supplementary Table 1 for descriptive statistics of the subject pool).

### Survey materials and procedure

In addition to various other measures outside the scope of the current paper, participants were given the seven-item CRT^45^, which consists of a reworded version of the original three-item CRT^25^ and a four-item non-numeric CRT^24^. For each subject, we calculated the CRT score as the proportion of correct answers to the CRT questions, resulting in a number [0–1]. Participants also completed a demographics questionnaire that included education, English fluency, social and economic political ideology (as separate questions), ethnicity, belief in God, religious affiliation, class, and income. We also recorded the time taken to complete the survey, which we follow ref. ^70^ in log transforming in our analysis because it has a highly right-skewed distribution.  Full experimental materials can be found here: https://osf.io/guk3m/.

### Twitter data

We then used the Twitter API to retrieve users’ public information, including general profile information (total number of tweets, accounts followed, followers, etc.), the content of their last 3200 tweets (capped by the Twitter API limit), and the list of accounts followed by each user in our dataset (only 6% of users in our sample happened to follow each other on Twitter). We retrieved data from Twitter on August 18, 2018. As part of our revisions during the peer review process, we also retrieved the tweets and retweets of all users on April 12, 2020 and merged the two datasets to maximize the number of tweets in our data. We linked the survey responses with Twitter data for our subsequent analysis.

For word-level analysis, we removed punctuation then cross-referenced all words in each tweet with the patterns in each word dictionary. We then flagged the tweet against all categories that had at least one pattern matched.

To create the co-follower network, we first constructed a bipartite graph representing all users in our study and all accounts they followed on Twitter. We then created the associated weighted mono-partite graph of the accounts that had at least *K* followers from our subject pool. Each account is represented by the aggregated demographic characteristics of its followers (e.g., fraction female, fraction US resident, fraction white, and average age).

### Reporting summary

Further information on research design is available in the Nature Research Reporting Summary linked to this article.

## Supplementary information

Supplementary Information

Reporting Summary

## Data Availability

For confidentiality reasons, the Twitter data are only available upon request. A reporting summary for this article is available as a Supplementary Information file. Full experimental materials can be found here: https://osf.io/guk3m/.
