# Supplementary material for: Cognitive reflection correlates with behavior on Twitter
Source: Nat Commun. 2021 Feb 10;12:921. doi: 10.1038/s41467-020-20043-0 (PMC7875970; doi:10.1038/s41467-020-20043-0)
Supplement: Supplementary file 1 — Supplementary Information [file 41467_2020_20043_MOESM1_ESM.pdf]

Supplementary Information

"Cognitive reflection correlates with behavior on Twitter"

Mosleh[1,2] et al.

1 SITE (Science, Innovation, Technology, and Entrepreneurship) Department, University of Exeter Business School, Exeter EX4 4PU, UK

2 Sloan School of Management, Massachusetts Institute of Technology, Cambridge, MA 02138

E-mail: mmosleh@mit.edu

1. Supplementary Figures

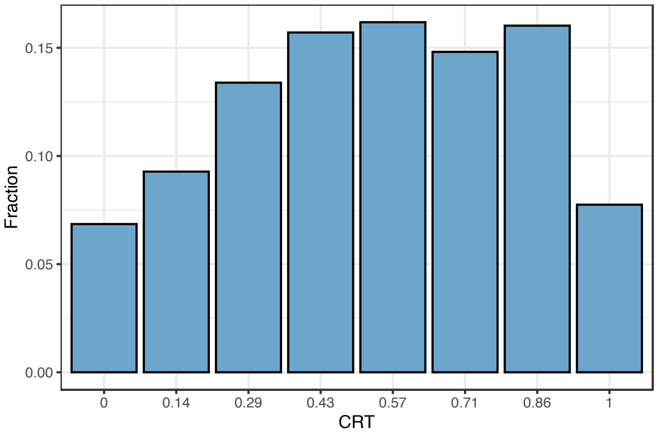

***Supplementary Figure 1. Distribution of users' CRT score in our sample.** Shown here is the distribution of proportion of correct answers given to the CRT questionnaire by individual subjects in our study.*

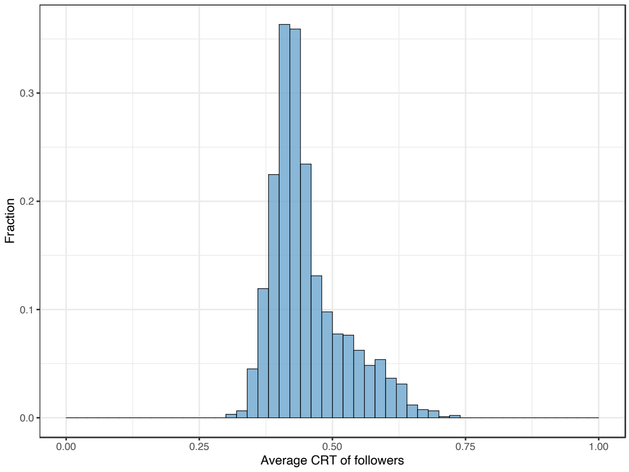

***Supplementary Figure 2. Distribution of average CRT score of followers in the co-follower network.** Shown here is the distribution of average CRT score of followers for each account in the co-follower network. This includes accounts followed by at least 25 users in our dataset (K=25).*

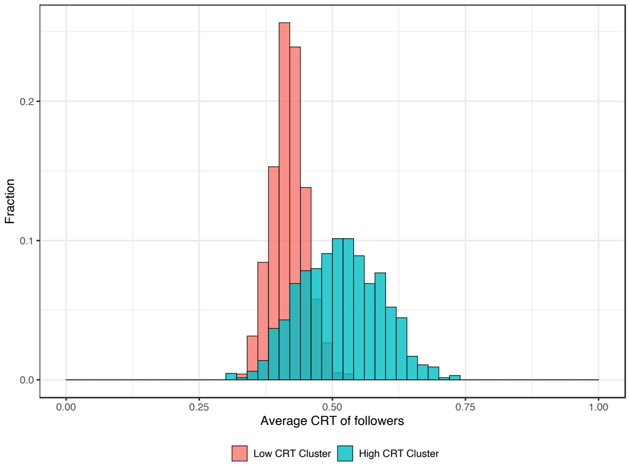

***Supplementary Figure 3. Distribution of average CRT of followers in clusters of the co-follower network.** Shown here are distributions of average CRT score of followers for two clusters within the co-follower network including accounts followed by at least 25 users in our dataset. There are one cluster of accounts that is mostly followed by users who scored lower in the CRT (narrower distribution) and one cluster of accounts that is mostly followed by users who scored mixed (both high and low) in the CRT (wider distribution).*

2. Supplementary Tables

Supplementary Table 1. Descriptive statistics for all variables. *Reported here are descriptive statistics of subjects' scores on the Cognitive Reflection Test, demographic information, and time to complete the survey.*

	Mean	*Median*	*STD*	*Min*	*Max*
CRT	.532	.571	.287	0	1
Age	34.128	33	11.224	18	66
Gender (female)	.554	1	.486	0	1
Ethnicity (white)	.834	1	.371	0	1
Political ideology (conservatism)	2.448	2.5	.918	1	5
US residency	.182	0	.386	0	1
Education (college degree)	.611	1	.488	0	1
Income	4.539	5	1.809	1	10
Log (time to complete survey)	6.673	6.645	.463	4.934	1.885

Supplementary Table 2. Correlation of (z-scored) covariates with CRT. *Reported here are coefficients and p-values from Pearson correlation two-sided test (results are not adjusted for multi-comparisons).*

	r	*p*
Age	-.076	**9.0e-04**
Gender (female)	-.106	**3.4e-06**
Ethnicity (white)	.058	**1.1e-02**
Political ideology (conservatism)	-.073	**1.5e-03**
US residency	<.001	.989
Education (college degree)	.105	**4.8e-06**
Income	.050	**2.9e-02**
Log (time to complete the survey)	.048	**3.8e-02**

Supplementary Table 3. Test for overdispersion of count data. *P-values are reported from Pearson's Chi-Squared test.*

	Mean	*Variance*	*Variance to mean ratio*	*Dispersion rate*	*p value*
Followed count	398.981	586,503.400	1,470.003	1,420.911	**0.0e+00**
Followers count	208.842	245,013.300	1,173.201	1,173.946	**0.0e+00**
Tweets count	4,014.138	294,920,100.000	73,470.359	62,713.881	**0.0e+00**
Favorites count	1,987.505	46,479,060.000	23,385.634	23,472.891	**0.0e+00**
Listed count	8.285	1,208.063	145.821	144.331	**0.0e+00**
Tweets in past two weeks	22.912	9,206.998	401.837	375.472	**0.0e+00**
Number of days on Twitter	2,355.463	1,146,860.000	486.894	486.970	**0.0e+00**

Supplementary Table 4. Followed count. *Results are generated using negative binominal regression taking users' z-scored CRT score as independent variable. Model 1) no controls; Model 2) controlling for age, gender, and ethnicity; Model 3) controlling for age, gender, ethnicity, US residency, education, social/economic conservatism, Income, and Log (time to complete the survey). p-values are reported based on two-tailed z-test and are also adjusted for multi-comparisons:* p_{BH} *is corrected p-value using Bonferroni-Holms and* p_{Holm} *using Benjamini Hochberg procedure.*

	Model 1				*Model 2*				*Model 3*			
	IRR (SE)	p	p_{BH}	p_{Holm}	IRR (SE)	p	p_{BH}	p_{Holm}	IRR (SE)	p	p_{BH}	p_{Holm}
CRT	.844 (.035)	**4.6e-05**	**3.2e-04**	**3.2e-04**	.867 (.035)	**4.3e-04**	**3.0e-03**	**3.0e-03**	.867 (.036)	**5.6e-04**	**3.9e-03**	**3.9e-03**
Age					1.168 (.044)	**3.5e-05**	**8.2e-05**	**1.8e-04**	1.189 (.045)	**5.7e-06**	**1.3e-05**	**2.8e-05**
Gender (female)					1.164 (.049)	**3.3e-04**	**5.8e-04**	**1.4e-03**	1.144 (.049)	**1.7e-03**	**4.0e-03**	**8.5e-03**
Ethnicity (white)					1.156 (.053)	**1.4e-03**	**3.1e-03**	**7.0e-03**	1.129 (.051)	**7.0e-03**	**1.2e-02**	**2.8e-02**
Political ideology (conservatism)									.954 (.046)	.332	.387	.876
US residency									.950 (.046)	.293	.684	1
Education (college degree)									1.011 (.043)	.796	.796	1
Income									.958 (.042)	.326	.456	.978
Log (time to complete the survey)									.966 (.048)	.481	.481	.752

Supplementary Table 5. Followers count. *Results are generated using negative binominal regression taking users' z-scored CRT score as independent variable. Model 1) no controls; Model 2) controlling for age, gender, and ethnicity; Model 3) controlling for age, gender, ethnicity, US residency, education, social/economic conservatism, Income, and Log (time to complete the survey). p-values are reported based on two-tailed z-test and are also adjusted for multi-comparisons:* p_{BH} *is corrected p-value using Bonferroni-Holms and* p_{Holm} *using Benjamini Hochberg procedure.*

	Model 1				*Model 2*				*Model 3*			
	IRR (SE)	p	p_{BH}	p_{Holm}	IRR (SE)	p	p_{BH}	p_{Holm}	IRR (SE)	p	p_{BH}	p_{Holm}
CRT	.956 (.049)	.375	.454	1	.985 (.048)	.752	.752	1	.965 (.045)	.447	.447	.96
Age					1.111 (.059)	**4.7e-02**	.066	.141	1.143 (.065)	**1.8e-02**	**3.2e-02**	.072
Gender (female)					1.155 (.065)	**1.0e-02**	**1.2e-02**	**2.0e-02**	1.129 (.059)	**2.1e-02**	**2.4e-02**	**4.2e-02**
Ethnicity (white)					1.182 (.063)	**1.8e-03**	**3.1e-03**	**7.2e-03**	1.176 (.062)	**2.0e-03**	**4.8e-03**	**1.0e-02**
Political ideology (conservatism)									.84 (.054)	**6.7e-03**	**1.6e-02**	**3.4e-02**
US residency									.984 (.064)	.798	.931	1
Education (college degree)									1.124 (.057)	**2.2e-02**	.084	.154
Income									1.018 (.049)	.719	.839	1
Log (time to complete the survey)									.965 (.046)	**3.9e-02**	.084	.222

Supplementary Table 6. Tweets count. *Results are generated using negative binominal regression taking users' z-scored CRT score as independent variable. Model 1) no controls; Model 2) controlling for age, gender, and ethnicity; Model 3) controlling for age, gender, ethnicity, US residency, education, social/economic conservatism, Income, and Log (time to complete the survey). p-values are reported based on two-tailed z-test and are also adjusted for multi-comparisons:* p_{BH} *is corrected p-value using Bonferroni-Holms and* p_{Holm} *using Benjamini Hochberg procedure.*

	Model 1				*Model 2*				*Model 3*			
	IRR (SE)	p	p_{BH}	p_{Holm}	IRR (SE)	p	p_{BH}	p_{Holm}	IRR (SE)	p	p_{BH}	p_{Holm}
CRT	.799 (.082)	**2.8e-02**	.072	.174	.878 (.079)	.147	.262	.735	.894 (.09)	.266	.341	.96
Age					1.004 (.067)	.957	.957	.957	1.05 (.081)	.522	.522	.522
Gender (female)					1.472 (.097)	**4.4e-09**	**3.1e-08**	**3.1e-08**	1.423 (.100)	**4.7e-07**	**2.9e-06**	**3.3e-06**
Ethnicity (white)					1.251 (.077)	**2.8e-04**	**9.7e-04**	**1.7e-03**	1.215 (.075)	**1.6e-03**	**4.8e-03**	**9.7e-03**
Political ideology (conservatism)									.849 (.087)	.108	.189	.432
US residency									.981 (.064)	.769	.931	1
Education (college degree)									.976 (.078)	.758	.796	1
Income									.887 (.067)	.111	.228	.600
Log (time to complete the survey)									.873 (.093)	.200	.280	.600

Supplementary Table 7. Favorites. *Results are generated using negative binominal regression taking users' z-scored CRT score as independent variable. Model 1) no controls; Model 2) controlling for age, gender, and ethnicity; Model 3) controlling for age, gender, ethnicity, US residency, education, social/economic conservatism, Income, and Log (time to complete the survey). p-values are reported based on two-tailed z-test and are also adjusted for multi-comparisons:* p_{BH} *is corrected p-value using Bonferroni-Holms and* p_{Holm} *using Benjamini Hochberg procedure.*

	Model 1				*Model 2*				*Model 3*			
	IRR (SE)	p	p_{BH}	p_{Holm}	IRR. (SE)	p	p_{BH}	p_{Holm}	IRR (SE)	p	p_{BH}	p_{Holm}
CRT	1.067 (.084)	.411	.454	1	1.096 (.084)	.236	.33	.735	1.099 (.082)	.204	.341	.96
Age					.792 (.085)	**3.1e-02**	.054	.124	.84 (.087)	.092	.129	.276
Gender (female)					1.357 (.114)	**2.7e-04**	**5.8e-04**	**1.4e-03**	1.269 (.1)	**2.6e-03**	**4.5e-03**	**1.0e-02**
Ethnicity (white)					1.007 (.086)	.939	.939	.939	1.022 (.079)	.782	.782	.782
Political ideology (conservatism)									.714 (.059)	**4.7e-05**	**3.3e-04**	**3.3e-04**
US residency									1.038 (.076)	.608	.931	1
Education (college degree)									.878 (.065)	.078	.182	.390
Income									.87 (.074)	.100	.228	.600
Log (time to complete the survey)									.882 (.056)	**4.8e-02**	.084	.222

Supplementary Table 8. Listed count. *Results are generated using negative binominal regression taking users' z-scored CRT score as independent variable. Model 1) no controls; Model 2) controlling for age, gender, and ethnicity; Model 3) controlling for age, gender, ethnicity, US residency, education, social/economic conservatism, Income, and Log (time to complete the survey). p-values are reported based on two-tailed z-test and are also adjusted for multi-comparisons:* p_{BH} *is corrected p-value using Bonferroni-Holms and* p_{Holm} *using Benjamini Hochberg procedure.*

	Model 1				*Model 2*				*Model 3*			
	IRR (SE)	p	p_{BH}	p_{Holm}	IRR (SE)	p	p_{BH}	p_{Holm}	IRR (SE)	p	p_{BH}	p_{Holm}
CRT	.902 (.1)	.352	.454	1	.954 (.091)	.617	.72	1	.891 (.098)	.292	.341	.96
Age					1.539 (.123)	**6.3e-08**	**2.2e-07**	**3.8e-07**	1.569 (.131)	**7.4e-08**	**2.6e-07**	**4.5e-07**
Gender (female)					1.433 (.137)	**1.7e-04**	**5.8e-04**	**1.0e-03**	1.464 (.113)	**8.4e-07**	**2.9e-06**	**5.1e-06**
Ethnicity (white)					1.5 (.131)	**3.2e-06**	**2.2e-05**	**2.2e-05**	1.546 (.127)	**1.1e-07**	**7.6e-07**	**7.6e-07**
Political ideology (conservatism)									.879 (.108)	.292	.387	.876
US residency									1.178 (.131)	.142	.497	.852
Education (college degree)									1.198 (.096)	**2.4e-02**	.084	.154
Income									1.015 (.089)	.864	.864	1
Log (time to complete the survey)									.885 (.122)	.376	.439	.752

Supplementary Table 9. Tweets in past two weeks. *Results are generated using negative binominal regression taking users' z-scored CRT score as independent variable. Model 1) no controls; Model 2) controlling for age, gender, and ethnicity; Model 3) controlling for age, gender, ethnicity, US residency, education, social/economic conservatism, Income, and Log (time to complete the survey). p-values are reported based on two-tailed z-test and are also adjusted for multi-comparisons:* p_{BH} *is corrected p-value using Bonferroni-Holms and* p_{Holm} *using Benjamini Hochberg procedure.*

	Model 1				*Model 2*				*Model 3*			
	IRR (SE)	p	p_{BH}	p_{Holm}	IRR (SE)	p	p_{BH}	p_{Holm}	IRR (SE)	p	p_{BH}	p_{Holm}
CRT	.811 (.079)	**3.1e-02**	.072	.174	.845 (.08)	.075	.262	.45	.865 (.096)	.192	.341	.96
Age					1.125 (.121)	.274	.32	.548	1.175 (.129)	.143	.167	.286
Gender (female)					1.238 (.12)	**2.8e-02**	**2.8e-02**	**2.8e-02**	1.155 (.109)	.125	.125	.125
Ethnicity (white)					1.186 (.106)	.056	.065	.112	1.144 (.096)	.11	.128	.22
Political ideology (conservatism)									.962 (.121)	.758	.758	.876
US residency									.83 (.062)	**1.2e-02**	.084	.084
Education (college degree)									.956 (.094)	.644	.796	1
Income									.746 (.067)	**1.1e-03**	**7.6e-03**	**7.6e-03**
Log (time to complete the survey)									.757 (.101)	**3.7e-02**	.084	.222

Supplementary Table 10. Days on Twitter. *Results are generated using negative binominal regression taking users' z-scored CRT score as independent variable. Model 1) no controls; Model 2) controlling for age, gender, and ethnicity; Model 3) controlling for age, gender, ethnicity, US residency, education, social/economic conservatism, Income, and Log (time to complete the survey). p-values are reported based on two-tailed z-test and are also adjusted for multi-comparisons:* p_{BH} *is corrected p-value using Bonferroni-Holms and* p_{Holm} *using Benjamini Hochberg procedure.*

	Model 1				*Model 2*				*Model 3*			
	IRR (SE)	p	p_{BH}	p_{Holm}	IRR (SE)	p	p_{BH}	p_{Holm}	IRR (SE)	p	p_{BH}	p_{Holm}
CRT	1.012 (.011)	.274	.458	1	1.02 (.011)	.072	.252	.432	1.016 (.011)	.135	.341	.81
Age					1.084 (.011)	**2.0e-16**	**1.4e-15**	**1.4e-15**	1.096 (.011)	**1.1e-19**	**7.8e-19**	**7.8e-19**
Gender (female)					1.038 (.011)	**7.3e-04**	**1.0e-03**	**2.2e-03**	1.033 (.011)	**3.3e-03**	**4.6e-03**	**1.0e-02**
Ethnicity (white)					1.037 (.013)	**4.5e-03**	**6.3e-03**	**1.3e-02**	1.031 (.013)	**1.6e-02**	**2.3e-02**	**4.9e-02**
Political ideology (conservatism)									.967 (.01)	**1.6e-03**	**5.7e-03**	**9.7e-03**
US residency									1.001 (.011)	.951	.951	1
Education (college degree)									1.01 (.011)	.358	.626	1
Income									.983 (.011)	.13	.228	.6
Log (time to complete the survey)									.962 (.011)	**9.5e-04**	**6.7e-03**	**6.7e-03**

Supplementary Table 11. Logistic regression predicting if the account belongs to low CRT cluster using the average characteristics of their followers in our sample (threshold of number of followers from our sample K=25). *Model 1) no controls; Model 2) controlling for age (average age of followers), gender (male fraction of followers), and ethnicity (white fraction of followers); Model 3) age (average age of followers), gender (male fraction of followers), and ethnicity (white fraction of followers), US residency (US resident fraction of followers), education (college degree fraction of followers), social/economic conservatism (average conservatism of followers), Income (average Income of followers), and average log (time to complete the survey) of followers. p-values are reported based on two-tailed z-test and without multi-comparisons adjustment.*

	Model 1		*Model 2*		*Model 3*	
	OR (SE)	*p*	*OR* (SE)	*p*	*OR* (SE)	*p*
(intercept)	2.038 (.068)	**0.0e+00**	.842 (.134)	.199	1.101 (.162)	.554
CRT	.090 (.117)	**0.0e+00**	.264 (.172)	**0.0e+00**	.545 (.208)	**3.5e-03**
Age			3.065 (.128)	**0.0e+00**	3.463 (.151)	**0.0e+00**
Gender (female)			28.168 (.279)	**0.0e+00**	16.38 (.294)	**0.0e+00**
Ethnicity (white)			2.145 (.192)	**6.7e-05**	1.014 (.239)	.953
Political ideology (conservatism)					1.308 (.185)	.146
US residency					.923 (.342)	.814
Education (college degree)					1.498 (.193)	**3.6e-02**
Income					.230 (.232)	**2.0e-10**
Log (time to complete the survey)					.332 (.157)	**0.0e+00**

Supplementary Table 12. Characteristics of clusters within co-followers' network for various threshold of number of followers K. *We included accounts in the network who had at least K followers from our sample.* ***.*** *Model 1) no controls; Model 2) controlling for age (average age of followers), gender (male fraction of followers), and ethnicity (white fraction of followers); Model 3) age (average age of followers), gender (male fraction of followers), and ethnicity (white fraction of followers), US residency (US resident fraction of followers), education (college degree fraction of followers), social/economic conservatism (average conservatism of followers), Income (average Income of followers), and average log (time to complete the survey) of followers. Across all values of the threshold for the number of followers, there exists one cluster with high average follower CRT score and one with low average follower CRT score. The average CRT of followers can significantly predict which of the two clusters the accounts belongs to. p-values are reported based on two-tailed z-test and without multi-comparisons adjustment (*p<.05, **p<.01, ***p<.001).*

**Threshold (*K*)**	**Cluster**	**CRT**		**Fraction of nodes in the cluster**	**Total nodes**	**Model 1 OR (SE)**	**Model 2 OR (SE)**	**Model 3 OR (SE)**
		**Mean**	**SD**					
30	Cluster 1	.52	.072	.379	1286	17.174*** (.164)	4.415*** (.238)	1.793* (.291)
	Cluster 2	.42	.029	.621	1286	.058*** (.164)	.227*** (.238)	.558* (.291)
25	Cluster 1	.515	.075	.35	1860	11.102*** (.117)	3.791*** (.172)	1.835** (.208)
	Cluster 2	.419	.032	.65	1860	.09*** (.117)	.264*** (.172)	.545** (.208)
20	Cluster 1	.517	.082	.316	2802	8.464*** (.085)	3.296*** (.119)	1.966*** (.142)
	Cluster 2	.422	.035	.237	2802	.523*** (.06)	.669*** (.095)	.524*** (.115)
	Cluster 3	.416	.037	.447	2802	.295*** (.063)	.562*** (.083)	.878 (.098)
15	Cluster 1	.512	.089	.314	4294	5.711*** (.058)	2.326*** (.079)	1.585*** (.095)
	Cluster 2	.419	.04	.243	4294	.539*** (.046)	.699*** (.069)	.559*** (.08)
	Cluster 3	.419	.046	.198	4294	.563*** (.049)	.814** (.06)	.844* (.073)
	Cluster 4	.414	.043	.245	4294	.462*** (.049)	.659*** (.069)	1.065 (.083)
10	Cluster 1	.512	.102	.338	7207	4.171*** (.038)	1.903*** (.053)	1.287*** (.063)
	Cluster 2	.418	.051	.233	7207	.57*** (.034)	.715*** (.047)	.648*** (.053)
	Cluster 3	.417	.054	.21	7207	.573*** (.036)	.887** (.044)	.904. (.052)
	Cluster 4	.415	.055	.22	7207	.538*** (.036)	.713*** (.048)	.978 (.056)

Supplementary Table 13. Predicting if the user tweeted from a news website using logistic regression. *Model 1) no controls; Model 2) controlling for age, gender, and ethnicity; Model 3) controlling for age, gender, ethnicity, US residency, education, social/economic conservatism, Income, and* Log (time to complete the survey). *p-values are reported based on z-test and without multi-comparisons adjustment.*

	Model 1		*Model 2*		*Model 3*	
	OR (SE)	*p*	*OR* (SE)	*p*	*OR* (SE)	*p*
(intercept)	.62 (.047)	**4.5e-24**	.612 (.048)	**1.7e-24**	.606 (.049)	**6.5e-25**
CRT	1.105 (.047)	**3.5e-02**	1.15 (.049)	**4.2e-03**	1.135 (.05)	**1.1e-02**
Age			1.304 (.049)	**4.9e-08**	1.389 (.051)	**1.1e-10**
Gender (female)			1.193 (.049)	**2.9e-04**	1.163 (.05)	**2.4e-03**
Ethnicity (white)			1.038 (.05)	.451	1.026 (.051)	.623
Political ideology (conservatism)					.784 (.05)	**1.4e-06**
US residency					1.006 (.049)	.902
Education (college degree)					1.076 (.051)	.149
Income					.96 (.051)	.422
Log (time to complete the survey)					.857 (.051)	**2.3e-03**

Supplementary Table 14. Predicting quality score of the tweeted outlet using linear regression with standard errors clustered on user. *Model 1) no control; Model 2) controlling for age, gender, and ethnicity; Model 3) controlling for age, gender, ethnicity, US residency, education, social/economic conservatism, Income, and* Log (time to complete the survey). *p-values are reported based on t-test and without multi-comparisons adjustment.*

	Model 1		*Model 2*		*Model 3*		*Model 3 + months dummies*	
	β (SE)	*p*	*β* (SE)	*p*	*β* (SE)	*p*	*β* (SE)	*p*
(intercept)	0 (.047)	1	0 (.044)	1	0 (.038)	1	.116 (.147)	.43
CRT	.101 (.04)	**9.7e-03**	.088 (.036)	**1.2e-02**	.09 (.036)	**1.2e-02**	.078 (.034)	**1.9e-02**
Age			-.061 (.043)	.157	-.018 (.041)	.66	-.013 (.038)	.734
Gender (female)			.014 (.043)	.75	.026 (.038)	.49	.001 (.031)	.987
Ethnicity (white)			.061 (.053)	.249	.005 (.044)	.904	-.004 (.04)	.929
Political ideology (conservatism)					-.181 (.049)	**1.9e-04**	-.177 (.046)	**1.2e-04**
US residency					-.091 (.041)	**2.6e-02**	-.078 (.036)	**3.0e-02**
Education (college degree)					.057 (.042)	.175	.055 (.039)	.163
Income					.017 (.039)	.657	.025 (.038)	.522
Log (time to complete the survey)					-.062 (.034)	.071	-.07 (.031)	**2.4e-02**
Month dummies	*No*		*No*		*No*		*Yes*	

Supplementary Table 15. Topic positively and negatively related to CRT vs. various number of topics in the model. *In all cases, there is always one topic (related to political engagement) that is positively correlated with CRT and one topic (related to "get rich quick" schemes) that is negatively related to CRT (p<.1, *p<.05, **p<.01, ***p<.001; p-values are reported based on t-test and without multi-comparisons adjustment.)*

Number of topics, *k*	Representative words for each topic	Coefficient of estimating topic proportion using CRT
5	**peopl, will, amp, can, just, like, say, trump, one, get, now, need, vote, time, make, think, year, know, right, want**	.10***
	**follow, amp, enter, giveaway, chanc, competit, end, retweet, winner, give, prize, simpli, just, freebiefriday, day, like, tampc, away, time**	-.076**
	just, get, like, one, love, can, time, day, now, dont, good, look, think, know, will, see, make, want, got, need	.071*
	via, just, thank, free, earn, new, get, check, today, amp, love, great, book, can, day, blog, use, now, make, sponsor	-.077 ***
	game, youtub, new, video, play, amp, watch, just, end, call, like, live, now, artist, onlin, one, top, playlist, wwe, run	-.021 (not sig.)
6	**peopl, will, amp, trump, just, say, can, like, vote, get, one, now, need, make, think, right, time, know, want, year**	.080**
	**follow, amp, enter, competit, chanc, giveaway, end, retweet, winner, give, simpli, freebiefriday, prize, like, just, day, tampc, away, good**	-.075**
	just, like, get, one, love, dont, time, can, now, know, day, think, good, want, peopl, look, make, got, will, see	.070*
	thank, day, amp, love, get, can, look, new, just, today, one, now, will, time, book, good, great, work, via, see	.010 (not sig.)
	youtub, game, new, video, thank, amp, music, like, follow, now, today, get, via, art, can, will, play, end, live, check	-.042*
	win, giveaway, enter, just, earn, free, chanc, via, gift, amp, card, check,get, sponsor, cash, prize, want, love, can, amazon	-.050*
7	**peopl, will, amp, trump, say, can, just, vote, like, now, one, get, need, right, make, think, time, brexit, want, know**	.076**
	**follow, amp, enter, competit, chanc, giveaway, end, retweet, winner, give, simpli, freebiefriday, prize, like, just, day, tampc, good, away**	-.064**
	get, just, will, now, day, today, one, good, time, look, can, amp, see, well, thank, love, year, back, last, week	.009 (not sig.)
	thank, via, book, amp, new, read, love, can, day, look, work, one, great, use, help, write, make, get, today, blog	<.001 (not sig.)
	just, like, get, one, love, dont, can, know, time, think, day, peopl, want, now, make, good, realli, thing, look, got	.066*
	youtub, game, new, video, like, music, follow, now, thank, get, play, amp, today, check, will, end, live, art, can, free	-.037·
	win, giveaway, enter, just, earn, free, chanc, via, amp, gift, card, check, sponsor, get, prize, cash, want, love, can, amazon	-.046*
8	**peopl, like, just, trump, will, one, say, can, get, know, make, dont, think, realdonaldtrump, want, time, need, thing, right, amp**	.054**
	**follow, amp, competit, enter, chanc, giveaway, end, retweet, winner, give, simpli, freebiefriday, prize, tampc, like, day, just, good, time**	-.066**
	amp, will, peopl, brexit, can, vote, now, pleas, get, one, say, just, year, need, time, think, like, support, nhs, make	.056**
	just, get, like, love, one, day, can, now, time, think, dont, look, good, know, got, need, work, want, will, today	.028 (not sig.)
	game, like, just, get, play, one, new, time, will, good, now, can, fuck, amp, watch, see, look, year, dont, end	.011 (not sig.)
	Thank, youtub, new, via, video, today, use, music, get, work, can, amp, great, follow, look, art, now, will, make, free	-.038*
	win, giveaway, enter, chanc, amp, free, just, gift, card, sponsor, check, follow, via, want, prize, get, can, game, now, new	-.020 (not sig.)
	just, via, book, earn, read, new, love, blog, review, write, thank, amp, watch, check, today, great, mile, day, get, post	-.017 (not sig.)
9	**peopl, will, amp, trump, say, vote, just, can, now, like, one, get, need, brexit, think, realdonaldtrump, right, make, time, year**	.064**
	**follow, amp, competit, enter, chanc, giveaway, end, retweet, winner, give, simpli, freebiefriday, prize, tampc, day, like, just, time, good**	-.070**
	thank, new, amp, can, work, use, today, great, get, will, make, music, time, one, look, now, art, write, help, read	.018 (not sig.)
	amp, game, will, get, team, win, play, one, today, good, see, look, great, just, time, fan, now, year, last, new	<.001 (not sig.)
	just, get, love, day, one, like, now, can, time, think, look, dont, good, will,got, know, need, today, work, thank	.011 (not sig.)
	like, just, get, one, dont, time, can, know, peopl, love, make, want, fuck, now, think, thing, good, day, realli, look	.067*
	via, amp, love, new, day, thank, sponsor, free, get, dog, can, look, great, make, help, tri, beauti, today, want, food	-.041*
	just, earn, via, book, check, watch, cash, read, review, free, mile, get, today, blog, onlin, call, topcashback, reward, prize, awesom	-.032*
	in, giveaway, enter, chanc, follow, youtub, amp, free, game, card, gift, just, video, end, retweet, like, new, now, can, contest	-.018 (not sig.)
10	**peopl, will, amp, trump, say, vote, just, can, now, like, one, get, need, brexit, think, right, realdonaldtrump, make, time, year**	.064**
	**follow, amp, competit, enter, chanc, giveaway, end, retweet, winner, give, simpli, freebiefriday, prize, tampc, like, day, just, time, good**	-.069**
	thank, new, use, can, work, today, amp, get, music, great, will, make, look, now, help, via, need, time, news, follow	.001 (not sig.)
	game, will, amp, get, win, team, play, good, today, fan, one, see, just, time, great, season, look, last, day, year	.001 (not sig.)
	book, read, via, new, amp, write, one, love, thank, just, day, review, blog, call, like, time, post, can, year, thing	.012 (not sig.)
	just, get, love, day, like, now, one, can, time, think, look, dont, good, will, got, today, need, work, know, thank	.064*
	via, amp, love, new, sponsor, day, thank, dog, free, get, help, can, make, great, look, tri, want, food, today, pleas	-.037*
	win, giveaway, enter, chanc, follow, amp, free, youtub, game, gift, card, just, retweet, end, new, like, prize, contest, winner, want	-.015 (not sig.)
	just, earn, check, via, cash, watch, free, get, mile, today, reward, topcashback, awesom, download, won, walk, prize, game, ipad, site	-.032*
	like, just, get, one, dont, time, can, know, peopl, love, make, want, fuck, now, think, thing, good, day, realli, look	.0178 *

Supplementary Table 16. Use of insight words and CRT. *Predicting use of LIWC word categories taking users' z-scored CRT score as independent variable using logistic regression at tweet-level with standard error cluster on username. Model 1) no controls; Model 2) controlling for age, gender, and ethnicity; Model 3) controlling for age, gender, ethnicity, US residency, education, social/economic conservatism, income, and Log (time to complete the survey). p-values are reported based on two-tailed z-test and are also adjusted for multi-comparisons: p_{BH} is corrected p-value using Bonferroni-Holms and p_{Holm} using Benjamini Hochberg procedure.*

	Model 1				*Model 2*				*Model 3*				*Model 3 + months dummies*			
	OR (SE)	*p*	p_{BH}	p_{Holm}	*OR* (SE)	*p*	p_{BH}	p_{Holm}	*OR* (SE)	*p*	p_{BH}	p_{Holm}	*OR* (SE)	*p*	p_{BH}	p_{Holm}
(intercept)	.134 (.026)	**0.0e+00**	**0.0e+00**	**0.0e+00**	.134 (.026)	**0.0e+00**	**0.0e+00**	**0.0e+00**	.133 (.025)	**0.0e+00**	**0.0e+00**	**0.0e+00**	.091 (.051)	**0.0e+00**	**0.0e+00**	**0.0e+00**
CRT	1.159 (.025)	**6.0e-09**	**4.2e-08**	**4.2e-08**	1.157 (.026)	**2.5e-08**	**1.7e-07**	**1.7e-07**	1.137 (.025)	**3.0e-07**	**2.1e-06**	**2.1e-06**	1.138 (.025)	**2.0e-07**	**1.4e-06**	**1.4e-06**
Age					.964 (.029)	.21	.245	.42	.979 (.028)	.458	.458	.76	.955 (.026)	.076	.089	.152
Gender (female)					.984 (.025)	.508	.593	1	.976 (.024)	.328	.459	1	.973 (.023)	.24	.42	.96
Ethnicity (white)					.979 (.018)	.249	.249	.249	.985 (.02)	.437	.437	.437	.985 (.018)	.388	.388	.388
Political ideology (conservatism)									.874 (.027)	**5.6e-07**	**2.0e-06**	**3.4e-06**	.880 (.027)	**1.7e-06**	**6.0e-06**	**1.0e-05**
US residency									1.029 (.021)	.17	.238	.51	1.032 (.02)	.115	.161	.345
Education (college degree)									1.046 (.026)	.079	.354	.553	1.05 (.025)	**.047**	.329	.329
Income									.992 (.027)	.759	.971	1	.995 (.026)	.835	.977	1
Log (time to complete the survey)									1.038 (.026)	.141	.197	.423	1.047 (.026)	.074	.13	.296
Months dummies	*No*				*No*				*No*				*Yes*			

Supplementary Table 17. Use of inhibition words and CRT. *Predicting use of LIWC word categories taking users' z-scored CRT score as independent variable using logistic regression at tweet-level with standard error cluster on username. Model 1) no controls; Model 2) controlling for age, gender, and ethnicity; Model 3) controlling for age, gender, ethnicity, US residency, education, social/economic conservatism, income, and Log (time to complete the survey). p-values are reported based on two-tailed z-test and are also adjusted for multi-comparisons:* p_{BH} *is corrected p-value using Bonferroni-Holms and* p_{Holm} *using Benjamini Hochberg procedure.*

	Model 1				*Model 2*				*Model 3*				*Model 3 + month dummies*			
	OR (SE)	p	p_{BH}	p_{Holm}	*OR* (SE)	p	p_{BH}	p_{Holm}	*OR* (SE)	p	p_{BH}	p_{Holm}	*OR* (SE)	p	p_{BH}	p_{Holm}
(intercept)	.072 (.031)	**0.0e+00**	**0.0e+00**	**0.0e+00**	.072 (.029)	**0.0e+00**	**0.0e+00**	**0.0e+00**	.071 (.029)	**0.0e+00**	**0.0e+00**	**0.0e+00**	0 (1.004)	**3.0e-25**	**3.0e-25**	**3.0e-25**
CRT	1.138 (.029)	**7.9e-06**	**2.5e-05**	**4.8e-05**	1.14 (.031)	**1.9e-05**	**6.5e-05**	**1.1e-04**	1.127 (.029)	**3.3e-05**	**1.2e-04**	**2.0e-04**	1.133 (.028)	**7.1e-06**	**1.9e-05**	**4.3e-05**
Age					1.124 (.035)	**9.5e-04**	**1.7e-03**	**3.8e-03**	1.141 (.034)	**1.1e-04**	**1.9e-04**	**4.3e-04**	1.1 (.031)	**2.1e-03**	**3.7e-03**	**8.5e-03**
Gender (female)					.997 (.031)	.918	.918	1	.997 (.033)	.919	.919	1	.991 (.03)	.759	.781	1
Ethnicity (white)					.958 (.02)	**2.9e-02**	**4.0e-02**	.087	.972 (.02)	.169	.197	.338	.966 (.018)	.062	.087	.186
Political ideology (conservatism)									.883 (.033)	**1.6e-04**	**3.3e-04**	**7.8e-04**	.893 (.032)	**4.1e-04**	**7.2e-04**	**1.8e-03**
US residency									1.077 (.024)	**2.4e-03**	**5.5e-03**	**1.2e-02**	1.074 (.023)	**2.2e-03**	**5.1e-03**	**1.1e-02**
Education (college degree)									.978 (.038)	.557	.886	1	.991 (.034)	.796	.923	1
Income									1.002 (.041)	.971	.971	1	1.006 (.039)	.873	.977	1
Log (time to complete the survey)									1.052 (.029)	.082	.144	.328	1.06 (.028)	**3.8e-02**	.089	.19
Months dummies	*No*				*No*				*No*				*Yes*			

Supplementary Table 18. Use of positive emotion words and CRT. *Predicting use of LIWC word categories taking users' z-scored CRT score as independent variable using logistic regression at tweet-level with standard error cluster on username. Model 1) no controls; Model 2) controlling for age, gender, and ethnicity; Model 3) controlling for age, gender, ethnicity, US residency, education, social/economic conservatism, income, and Log (time to complete the survey). p-values are reported based on two-tailed z-test and are also adjusted for multi-comparisons:* p_{BH} *is corrected p-value using Bonferroni-Holms and* p_{Holm} *using Benjamini Hochberg procedure.*

	Model 1				*Model 2*				*Model 3*				*Model 3 + month dummies*			
	OR (SE)	*p*	p_{BH}	p_{Holm}	*OR* (SE)	*p*	p_{BH}	p_{Holm}	*OR* (SE)	*p*	p_{BH}	p_{Holm}	*OR* (SE)	*p*	p_{BH}	p_{Holm}
(intercept)	1.133 (.032)	**7.7e-05**	**7.7e-05**	**7.7e-05**	1.134 (.029)	**1.9e-05**	**1.9e-05**	**1.9e-05**	1.135 (.029)	**9.1e-06**	**9.1e-06**	**9.1e-06**	.063 (.06)	**0.0e+00**	**0.0e+00**	**0.0e+00**
CRT	.915 (.033)	**7.0e-03**	**8.2e-03**	**1.4e-02**	.941 (.032)	.054	.063	.108	.962 (.031)	.217	.253	.434	.966 (.029)	.235	.235	.46
Age					1.141 (.029)	**6.4e-06**	**4.5e-05**	**4.5e-05**	1.139 (.028)	**3.7e-06**	**8.6e-06**	**2.1e-05**	1.118 (.028)	**8.8e-05**	**3.1e-04**	**5.3e-04**
Gender (female)					1.213 (.025)	**1.7e-14**	**1.2e-13**	**1.2e-13**	1.203 (.025)	**2.8e-13**	**1.9e-12**	**1.9e-12**	1.200 (.025)	**2.5e-13**	**1.7e-12**	**1.7e-12**
Ethnicity (white)					1.093 (.019)	**2.5e-06**	**1.7e-05**	**1.7e-05**	1.073 (.019)	**2.8e-04**	**1.0e-03**	**2.0e-03**	1.068 (.02)	**9.8e-04**	**3.4e-03**	**5.9e-03**
Political ideology (conservatism)									1.074 (.031)	**1.9e-02**	**2.2e-02**	**3.8e-02**	1.079 (.03)	**1.1e-02**	**1.3e-02**	**2.1e-02**
US residency									.93 (.022)	**1.2e-03**	**4.4e-03**	**7.5e-03**	.924 (.022)	**3.1e-04**	**1.1e-03**	**1.8e-03**
Education (college degree)									.954 (.029)	.101	.354	.606	.961 (.028)	.152	.532	.912
Income									.938 (.028)	**2.4e-02**	.168	.168	.945 (.027)	**4.0e-02**	.14	.24
Log (time to complete the survey)									.931 (.03)	**1.9e-02**	.066	.114	.935 (.028)	**1.7e-02**	.06	.102
Months dummies	*No*				*No*				*No*				*Yes*			

Supplementary Table 19. Use of negative emotion words and CRT. *Predicting use of LIWC word categories taking users' z-scored CRT score as independent variable using logistic regression at tweet-level with standard error cluster on username. Model 1) no controls; Model 2) controlling for age, gender, and ethnicity; Model 3) controlling for age, gender, ethnicity, US residency, education, social/economic conservatism, income, and Log (time to complete the survey). p-values are reported based on two-tailed z-test and are also adjusted for multi-comparisons:* p_{BH} *is corrected p-value using Bonferroni-Holms and* p_{Holm} *using Benjamini Hochberg procedure.*

	Model 1				*Model 2*				*Model 3*				*Model 3 + month dummies*			
	OR (SE)	*p*	p_{BH}	p_{Holm}	*OR* (SE)	*p*	p_{BH}	p_{Holm}	*OR* (SE)	*p*	p_{BH}	p_{Holm}	*OR* (SE)	*p*	p_{BH}	p_{Holm}
(intercept)	.162 (.029)	**0.0e+00**	**0.0e+00**	**0.0e+00**	.161 (.03)	**0.0e+00**	**0.0e+00**	**0.0e+00**	.16 (.029)	**0.0e+00**	**0.0e+00**	**0.0e+00**	.134 (.055)	**4.0e-294**	**5.6e-294**	**1.2e-293**
CRT	1.142 (.03)	**1.1e-05**	**2.5e-05**	**5.5e-05**	1.137 (.031)	**2.8e-05**	**6.5e-05**	**1.4e-04**	1.118 (.029)	**1.3e-04**	**2.3e-04**	**5.3e-04**	1.124 (.029)	**5.2e-05**	**9.1e-05**	**2.1e-04**
Age					.935 (.035)	.052	.073	.156	.956 (.034)	.181	.253	.543	.933 (.031)	**2.8e-02**	**3.9e-02**	.084
Gender (female)					.962 (.028)	.16	.373	.8	.956 (.027)	.099	.231	.495	.953 (.026)	.064	.149	.32
Ethnicity (white)					.948 (.02)	**8.5e-03**	**1.5e-02**	**3.4e-02**	.955 (.021)	**3.2e-02**	.056	.128	.954 (.02)	**1.8e-02**	**3.1e-02**	.072
Political ideology (conservatism)									.856 (.03)	**2.8e-07**	**2.0e-06**	**2.0e-06**	.862 (.03)	**6.3e-07**	**4.4e-06**	**4.4e-06**
US residency									1.054 (.025)	**3.8e-02**	.066	.152	1.056 (.024)	**2.4e-02**	**4.3e-02**	.096
Education (college degree)									1.009 (.029)	.759	.886	1	1.013 (.028)	.64	.923	1
Income									.994 (.03)	.852	.971	1	.999 (.029)	.977	.977	1
Log (time to complete the survey)									1.027 (.028)	.34	.397	.68	1.033 (.028)	.259	.302	.518
Months dummies	*No*				*No*				*No*				*Yes*			

Supplementary Table 20. Use of moral words and CRT. *Predicting use of LIWC word categories taking users' z-scored CRT score as independent variable using logistic regression at tweet-level with standard error cluster on username. Model 1) no controls; Model 2) controlling for age, gender, and ethnicity; Model 3) controlling for age, gender, ethnicity, US residency, education, social/economic conservatism, income, and Log (time to complete the survey). p-values are reported based on two-tailed z-test and are also adjusted for multi-comparisons: p_{BH} is corrected p-value using Bonferroni-Holms and p_{Holm} using Benjamini Hochberg procedure.*

	Model 1				*Model 2*				*Model 3*				*Model 3 + month dummies*			
	OR (SE)	*p*	p_{BH}	p_{Holm}	*OR* (SE)	*p*	p_{BH}	p_{Holm}	*OR* (SE)	*p*	p_{BH}	p_{Holm}	*OR* (SE)	*p*	p_{BH}	p_{Holm}
(intercept)	.268 (.02)	**0.0e+00**	**0.0e+00**	**0.0e+00**	.267 (.019)	**0.0e+00**	**0.0e+00**	**0.0e+00**	.266 (.018)	**0.0e+00**	**0.0e+00**	**0.0e+00**	.189 (.036)	**0.0e+00**	**0.0e+00**	**0.0e+00**
CRT	1.072 (.018)	**1.1e-04**	**1.9e-04**	**4.3e-04**	1.079 (.018)	**4.4e-05**	**7.7e-05**	**1.8e-04**	1.074 (.018)	**8.9e-05**	**2.1e-04**	**4.5e-04**	1.078 (.017)	**8.0e-06**	**1.9e-05**	**4.3e-05**
Age					1.097 (.022)	**2.9e-05**	**6.8e-05**	**1.5e-04**	1.108 (.022)	**3.4e-06**	**8.6e-06**	**2.1e-05**	1.081 (.021)	**1.7e-04**	**3.9e-04**	**8.4e-04**
Gender (female)					1.025 (.021)	.227	.397	.908	1.022 (.021)	.317	.459	1	1.018 (.02)	.366	.512	1
Ethnicity (white)					.964 (.019)	.059	.069	.118	.967 (.021)	.101	.141	.303	.964 (.021)	.075	.088	.186
Political ideology (conservatism)									.938 (.02)	**1.1e-03**	**1.6e-03**	**3.4e-03**	.945 (.018)	**2.0e-03**	**2.8e-03**	**5.9e-03**
US residency									1.024 (.021)	.254	.296	.51	1.022 (.02)	.272	.317	.544
Education (college degree)									1.003 (.02)	.895	.895	1	1.012 (.019)	.533	.923	1
Income									.981 (.023)	.408	.871	1	.989 (.021)	.594	.977	1
Log (time to complete the survey)									1.008 (.019)	.666	.666	.68	1.015 (.018)	.412	.412	.518
Months dummies	*No*				*No*				*No*				*Yes*			

***Supplementary Table 21*. Use of political words and CRT.** *Predicting use of LIWC word categories taking users' z-scored CRT score as independent variable using logistic regression at tweet-level with standard error cluster on username. Model 1) no controls; Model 2) controlling for age, gender, and ethnicity; Model 3) controlling for age, gender, ethnicity, US residency, education, social/economic conservatism, income, and Log (time to complete the survey). p-values are reported based on two-tailed z-test and are also adjusted for multi-comparisons: p_{BH} is corrected p-value using Bonferroni-Holms and p_{Holm} using Benjamini Hochberg procedure.*

	Model 1				*Model 2*				*Model 3*				*Model 3 + month dummies*			
	OR (SE)	*p*	p_{BH}	p_{Holm}	*OR* (SE)	*p*	p_{BH}	p_{Holm}	*OR* (SE)	*p*	p_{BH}	p_{Holm}	*OR* (SE)	*p*	p_{BH}	p_{Holm}
(intercept)	.092 (.06)	**0.0e+00**	**0.0e+00**	**0.0e+00**	.088 (.054)	**0.0e+00**	**0.0e+00**	**0.0e+00**	.083 (.054)	**0.0e+00**	**0.0e+00**	**0.0e+00**	.118 (.112)	**6.3e-81**	**7.4e-81**	**1.3e-80**
CRT	1.193 (.055)	**1.3e-03**	**1.9e-03**	**4.0e-03**	1.177 (.06)	**6.4e-03**	**8.9e-03**	**1.9e-02**	1.157 (.057)	**1.0e-02**	**1.4e-02**	**3.1e-02**	1.167 (.056)	**5.6e-03**	**7.8e-03**	**1.7e-02**
Age					1.333 (.066)	**1.5e-05**	**5.2e-05**	**9.0e-05**	1.371 (.064)	**8.4e-07**	**5.8e-06**	**5.8e-06**	1.329 (.062)	**3.7e-06**	**2.6e-05**	**2.6e-05**
Gender (female)					.884 (.062)	**4.7e-02**	.164	.282	.889 (.061)	.055	.192	.33	.882 (.059)	**3.3e-02**	.116	.198
Ethnicity (white)					.867 (.047)	**2.3e-03**	**5.4e-03**	**1.4e-02**	.909 (.042)	**2.3e-02**	.054	.115	.9 (.041)	**1.1e-02**	**2.6e-02**	.055
Political ideology (conservatism)									.764 (.072)	**1.9e-04**	**3.3e-04**	**7.8e-04**	.777 (.071)	**3.7e-04**	**7.2e-04**	**1.8e-03**
US residency									1.266 (.054)	**1.3e-05**	**9.4e-05**	**9.4e-05**	1.245 (.053)	**3.3e-05**	**2.3e-04**	**2.3e-04**
Education (college degree)									.976 (.062)	.693	.886	1	.994 (.059)	.923	.923	1
Income									.961 (.06)	.498	.871	1	.97 (.057)	.591	.977	1
Log (time to complete the survey)									1.142 (.047)	**5.0e-03**	**3.5e-02**	**3.5e-02**	1.153 (.046)	**2.1e-03**	**1.5e-02**	**1.5e-02**
Months dummies	*No*				*No*				*No*				*Yes*			

Supplementary Table 22. Use of political words and political extremity (distance from the scale midpoint for the partisanship measure). *Predicting use of LIWC word categories taking users' political extermity independent variable using logistic regression at tweet-level with standard error cluster on username. Model 1) no controls; Model 2) controlling for age, gender, and ethnicity; Model 3) controlling for age, gender, ethnicity, US residency, education, social/economic conservatism, income, and Log (time to complete the survey). p-values are reported based on two-tailed z-test.*

	Model 1		*Model 2*		*Model 3*		*Model 3 + month dummies*	
	OR (SE)	*p*	*OR* (SE)	*p*	*OR* (SE)	*p*	*OR* (SE)	*p*
(intercept)	.089 (.062)	**0.0e+00**	.085 (.055)	**0.0e+00**	.082 (.052)	**0.0e+00**	.099 (.116)	**4.3e-88**
Political extremity	1.341 (.059)	**6.9e-07**	1.382 (.058)	**2.8e-08**	1.25 (.093)	**1.7e-02**	1.235 (.09)	**1.9e-02**
Age			1.348 (.064)	**2.8e-06**	1.346 (.066)	**6.9e-06**	1.312 (.063)	**1.7e-05**
Gender (female)			.844 (.056)	**2.6e-03**	.859 (.059)	**1.1e-02**	.85 (.058)	**4.8e-03**
Ethnicity (white)			.874 (.047)	**4.0e-03**	.916 (.043)	**3.8e-02**	.907 (.043)	**2.2e-02**
Political ideology (conservatism)					.877 (.095)	.169	.881 (.093)	.172
US residency					1.245 (.056)	**8.0e-05**	1.226 (.054)	**1.8e-04**
Education (college degree)					.976 (.063)	.703	.992 (.06)	.899
Income					.966 (.061)	.57	.978 (.058)	.701
Log (time to complete the survey)					1.15 (.048)	**3.5e-03**	1.161 (.047)	**1.5e-03**
Months dummies	*No*		*No*		*No*		*Yes*	
