# Supplementary material for: Cognitive reflection correlates with behavior on Twitter
Source: Nat Commun. 2021 Feb 10;12:921. doi: 10.1038/s41467-020-20043-0 (PMC7875970; doi:10.1038/s41467-020-20043-0)
Supplement: Supplementary file 2 — Reporting Summary [file 41467_2020_20043_MOESM2_ESM.pdf]

Corresponding author(s): Mohsen Mosleh

Last updated by author(s): Aug 27, 2020

Reporting Summary

Nature Research wishes to improve the reproducibility of the work that we publish. This form provides structure for consistency and transparency in reporting. For further information on Nature Research policies, see our Editorial Policies and the Editorial Policy Checklist.

Statistics

For all statistical analyses, confirm that the following items are present in the figure legend, table legend, main text, or Methods section.

n/a	Confirmed	
☐	☒	The exact sample size (*n*) for each experimental group/condition, given as a discrete number and unit of measurement
☒	☐	A statement on whether measurements were taken from distinct samples or whether the same sample was measured repeatedly
☐	☒	The statistical test(s) used AND whether they are one- or two-sided<br>*Only common tests should be described solely by name; describe more complex techniques in the Methods section.*
☐	☒	A description of all covariates tested
☐	☒	A description of any assumptions or corrections, such as tests of normality and adjustment for multiple comparisons
☐	☒	A full description of the statistical parameters including central tendency (e.g. means) or other basic estimates (e.g. regression coefficient) AND variation (e.g. standard deviation) or associated estimates of uncertainty (e.g. confidence intervals)
☐	☒	For null hypothesis testing, the test statistic (e.g. *F*, *t*, *r*) with confidence intervals, effect sizes, degrees of freedom and *P* value noted<br>*Give P values as exact values whenever suitable.*
☒	☐	For Bayesian analysis, information on the choice of priors and Markov chain Monte Carlo settings
☒	☐	For hierarchical and complex designs, identification of the appropriate level for tests and full reporting of outcomes
☐	☒	Estimates of effect sizes (e.g. Cohen's *d*, Pearson's *r*), indicating how they were calculated

Our web collection on statistics for biologists contains articles on many of the points above.

Software and code

Policy information about availability of computer code

Data collection	Data was collected using Python 3.6
Data analysis	All statistical analysis was done using R 3.6.6

For manuscripts utilizing custom algorithms or software that are central to the research but not yet described in published literature, software must be made available to editors and reviewers. We strongly encourage code deposition in a community repository (e.g. GitHub). See the Nature Research guidelines for submitting code & software for further information.

Data

Policy information about availability of data

All manuscripts must include a data availability statement. This statement should provide the following information, where applicable:

- Accession codes, unique identifiers, or web links for publicly available datasets
- A list of figures that have associated raw data
- A description of any restrictions on data availability

For confidentiality reasons the Twitter data are only available upon request.

Field-specific reporting

Please select the one below that is the best fit for your research. If you are not sure, read the appropriate sections before making your selection.

☐ Life sciences ☒ Behavioural & social sciences ☐ Ecological, evolutionary & environmental sciences

For a reference copy of the document with all sections, see nature.com/documents/nr-reporting-summary-flat.pdf

Behavioural & social sciences study design

All studies must disclose on these points even when the disclosure is negative.

Study description	We investigate the relationship between individuals difference in cognitive reflection and Online behavior on Twitter using quantitative data we collected through a survey study combined with Twitter data.
Research sample	We recruited participants via Prolific, a subject pool for online experiments that consists of mostly UK- and US-based individuals. We used a feature on Prolific to selectively recruit participants who self-reported using Twitter on a regular basis. We had complete data and usable Twitter IDs for 1,901 users (55% female, Median_age = 33, 43% UK residents, 18% US residents, and the rest mostly from Canada, Spain, Italy, and Portugal. This sample is based on Prolific participants who self-reported to have Twitter ID; thus may not be representative of Twitter users in general. Testing the generalizablity of results is a direction for future research .
Sampling strategy	We used a feature on Prolific to selectively recruit participants who self-reported using Twitter on a regular basis. We recruited participants from June 15, 2018, to June 20, 2018. Twitter IDs were provided by participants at the beginning of the study.
Data collection	We collected individuals' data using Qualtrics survey recruiting subjects from Prolific and also used Twitter API to collect data from individuals public profile. In the survey, we asked subjects for the demographic information, asked them about accuracy of a set of false and true headlines, and also to do the Cognitive Reflection Test.
Timing	We recruited 2,010 participants in five days (from June 15, 2018, to June 20, 2018). We used Twitter API to pull users' public information (accounts followed, profile characteristics, and content of tweets) from Twitter on August 18 2018. We also did another data pull on April 12 2020 for the content of tweets and merged the content from the previous data pull. (Our results were robust to the additional data)
Data exclusions	Some participants entered obviously fake Twitter IDs – for example, the accounts of celebrities. To screen out such accounts, we excluded accounts with follower counts above the 95th percentile in our dataset.
Non-participation	Of 2,010 participants recruited, 1,901 individuals had complete data and usable Twitter IDs. Thus 109 individuals dropped out either because they did not complete the survey or provided invalid Twitter ID.
Randomization	This is was a correlational study thus we did not do randomization to different experimental conditions. All subjects went through the same survey questions to identify individual differences (demographics, cognitive reflection). We then invesiateged how those individual differences relates to their online behavior.

Reporting for specific materials, systems and methods

We require information from authors about some types of materials, experimental systems and methods used in many studies. Here, indicate whether each material, system or method listed is relevant to your study. If you are not sure if a list item applies to your research, read the appropriate section before selecting a response.

Materials & experimental systems

n/a	Involved in the study	
☒	☐	Antibodies
☒	☐	Eukaryotic cell lines
☒	☐	Palaeontology and archaeology
☒	☐	Animals and other organisms
☐	☒	Human research participants
☒	☐	Clinical data
☒	☐	Dual use research of concern

Methods

n/a	Involved in the study	
☒	☐	ChIP-seq
☒	☐	Flow cytometry
☒	☐	MRI-based neuroimaging

Human research participants

Policy information about studies involving human research participants

Population characteristics	55% female, Median_age = 33, 43% UK residents, 18% US residents and the rest mostly from Canada, Spain, Italy, and Portugal

Recruitment	Subjects were recruited from Prolific and to those who self-reported to have Twitter accounts
Ethics oversight	Our studies were approved by the Yale Human Subjects Committee , IRB Protocol # 2000022539

Note that full information on the approval of the study protocol must also be provided in the manuscript.
